# CRISPR‐Cas9 and precision editing technologies linking functional genomics to clinical translation in genetic diseases

**DOI:** 10.1002/ctm2.70764

**Published:** 2026-09-24

**Authors:** Zijing Wen, Jianming Su

**Affiliations:** ^1^ College of Animal Medicine Hunan Agricultural University Changsha China

**Keywords:** clinical translation, CRISPR‐Cas9, gene function research, genetic diseases, pathogenic mutations, precision editing

## Abstract

**Background:**

CRISPR‐Cas9 and derivative precision‐editing platforms increasingly connect pathogenic variant interpretation with functional genomics and therapeutic development in genetic diseases. This narrative review focuses on a variant‐mechanism‐driven framework for matching editing strategies to mutation structure, functional consequence, disease‐model evidence, delivery feasibility, safety risk, and translational readiness.

**Main body:**

The review summarizes how monogenic, polygenic, coding, non‐coding, mitochondrial, and complex disease contexts influence the choice of canonical Cas9 editing, base editing, prime editing, Cas variants, CRISPR interference/activation, epigenome editing, and disease‐model systems. It further compares ex vivo and in vivo delivery routes, safety assessment strategies, immunogenicity and genotoxicity concerns, and clinical implementation barriers, including CMC/manufacturing scalability, long‐term follow‐up, affordability, and regulatory oversight. Current evidence supports the clinical maturity of ex vivo hematopoietic editing, whereas most in vivo and precision‐repair approaches remain constrained by delivery, durability, product heterogeneity, and safety uncertainties.

**Conclusion:**

The central conclusion is that future CRISPR‐based interventions should be judged not only by editability, but by whether molecular correction can be translated into durable, safe, manufacturable, and clinically meaningful benefit.

## Highlights

- A variant‐mechanism‐driven framework can guide the choice of CRISPR editing strategy for diverse genetic diseases.
- Ex vivo hematopoietic editing currently shows the clearest clinical maturity among CRISPR‐based interventions.
- In vivo and precision‐repair approaches remain limited by delivery, durability, product heterogeneity, and safety uncertainties.
- Clinical translation requires integrated consideration of safety, manufacturability, regulatory alignment, and equitable access.

## Graphical abstract

CRISPR‐Cas9 and precision editing technologies enable a variant‐mechanism‐driven framework for genetic disease research and therapeutic development. Pathogenic variants are first interpreted according to mutation type, coding or regulatory consequence, tissue context, and disease mechanism. Editing tools, including nuclease‐mediated editing, base editing, prime editing, CRISPRi/a, and epigenome editing, can then be matched to specific repair or functional‐modulation goals. Cellular models, iPSCs, organoids, and animal models provide mechanistic validation and phenotypic rescue evidence. Clinical translation further requires appropriate delivery platforms, multi‐modal safety assessment, manufacturability, regulatory alignment, and real‐world access strategies.

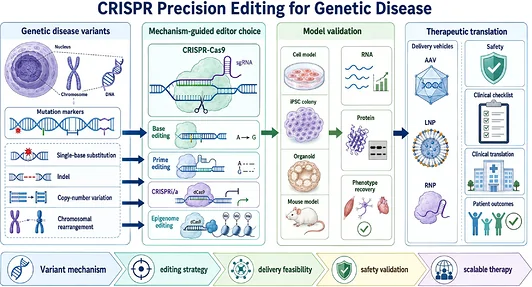

## 1 INTRODUCTION

The etiological intervention of genetic diseases—encompassing monogenic diseases, polygenic diseases, chromosomal abnormalities, and complex genetic susceptibility disorders—directly targets the genetic level within precision medicine, as their pathological origin can usually be traced to disruptions in gene sequence, dosage, or regulation.^1^ Rare diseases affect approximately 300 million people worldwide, with over 6000 clinically defined rare diseases identified; notably, about 72% have a genetic basis and approximately 70% have an onset in childhood.^2^ Faced with frequent diagnostic delays, prolonged disease burden, and scarce therapeutic options, research has increasingly focused on building mechanistic explanations and precision interventions directly from the pathogenic mutations themselves.^3^

Pathogenic mutations exert their effects at the molecular level not as simple deoxyribonucleic acid (DNA) sequence alterations, but by disrupting protein architecture, ribonucleic acid (RNA) splicing, transcriptional programs, and broader signalling networks.^4^, ^5^ Functional genomics evidence demonstrates that loss‐of‐function mutations can diminish protein expression or activity, whereas gain‐of‐function mutations may drive aberrant pathway activation, and dominant‐negative mutations can antagonize the products of the normal allele. Furthermore, studies of non‐coding variants and regulatory element anomalies underscore that restricting causal explanations to coding region changes is insufficient for many genetic diseases. A coherent mechanistic framework therefore requires assembling a continuous evidential chain—from pathogenic mutation, through gene function imbalance and cellular state disruption, to the altered tissue phenotype.

While traditional therapies and conventional gene function research methods have provided an essential foundation, their constraints are becoming increasingly clear under the lens of precision medicine.^6^ Protein replacement, metabolic modulation, and symptom control can alleviate clinical manifestations in certain genetic diseases, yet they rarely correct the pathogenic mutation itself. Methodological evaluations indicate that RNA interference may suffer from incomplete knockdown and non‐specific off‐target activity, and that conventional overexpression systems and non‐isogenic models frequently fail to replicate endogenous expression levels and patient‐specific genetic backgrounds. These limitations create an urgent demand for technological platforms that can precisely mimic, repair, or modulate pathogenic mutations at the endogenous locus, fuelling both mechanistic investigation and therapeutic development.

Responding to these demands, CRISPR‐Cas9 technology opens a new avenue for functional validation at the endogenous locus.^7^, ^8^ The system employs guide RNA to direct Cas nucleases to specific target sequences, generating site‐specific edits applicable to gene knockout, mutation modelling, targeted knock‐in, and disease model construction. Consequently, genetic disease research is progressively transitioning from correlational descriptions towards causally explanatory functional validation. The subsequent emergence of base editing, prime editing,^6^, ^7^ Cas protein variants, and non‐double‐strand break regulation strategies has further broadened the scope for repairing diverse pathogenic mutation types with the CRISPR platform. Clinically, the Food and Drug Administration (FDA) has approved the first cell and gene therapy product employing CRISPR‐Cas9 technology for sickle cell disease and β‐thalassemia, signalling that precision editing has entered real‐world application for hemoglobinopathies, though broader applicability remains limited by delivery efficiency, safety evaluation, and treatment accessibility.

**FIGURE 1:**
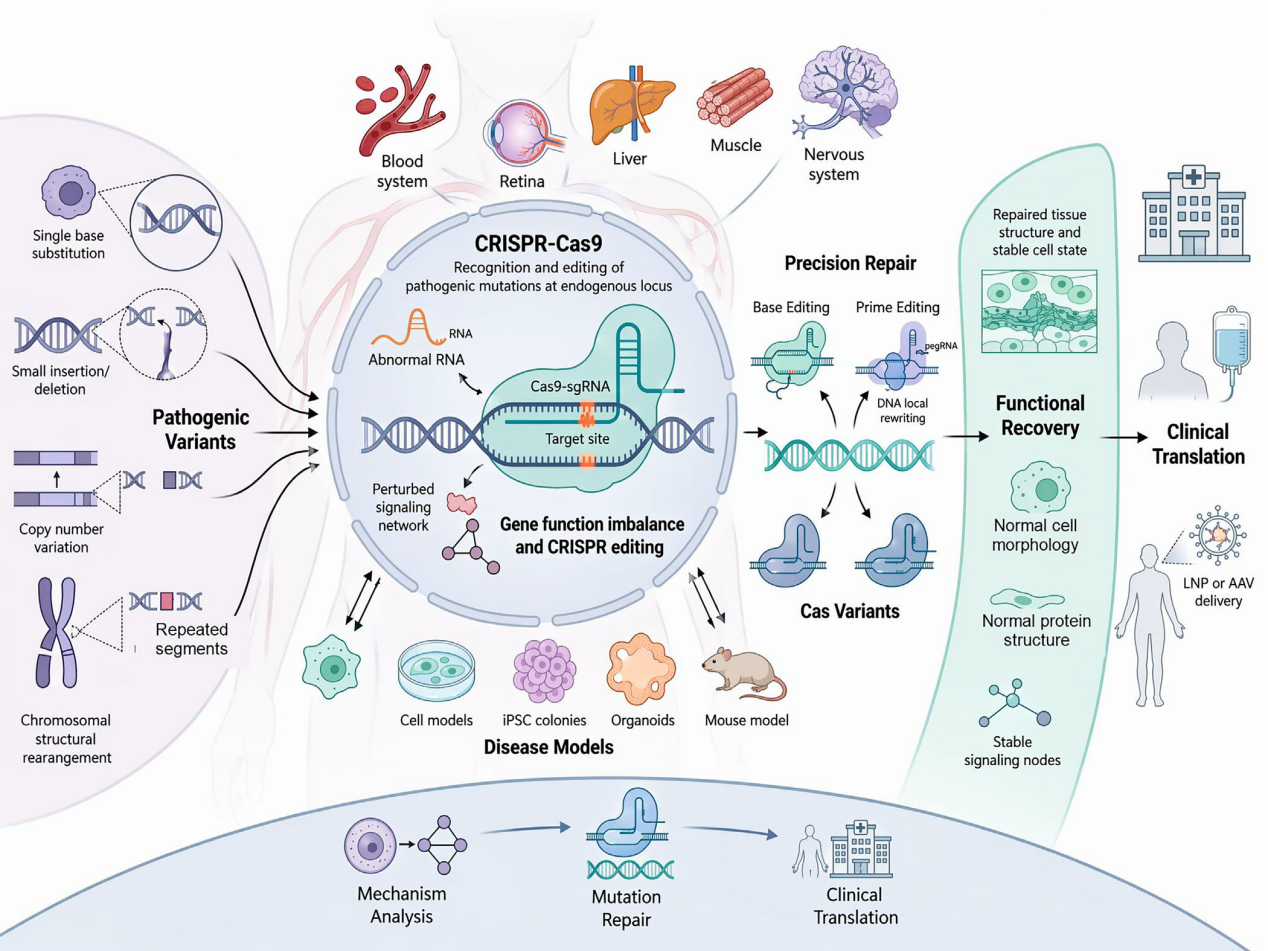
Overall framework of CRISPR precision editing driving the elucidation of genetic disease mechanisms and mutation correction.

Collectively, the role of CRISPR‐Cas9 in genetic diseases can be framed as an analytical thread linking mechanistic dissection to precision repair: characterize how pathogenic mutations produce gene function imbalance, validate the causal mutation–phenotype relationship, and then develop mutation‐repair strategies guided by appropriate editing tools, delivery systems, and safety assessments. Rather than being reduced to a gene‐cutting tool, CRISPR‐Cas9 is more accurately viewed as a systematic technology platform that integrates pathogenic mutation identification, gene function verification, disease model construction, functional restoration evaluation, and clinical translation. This framework reflects the current organization and developmental trajectory of research progress, without implying uniform maturity across all steps or universal clinical translatability (Figure 1).

Review scope and literature‐selection strategy. This narrative review asks how CRISPR‐based editing strategies should be matched to pathogenic variant mechanisms, disease‐model evidence, delivery feasibility, safety risks, and translational readiness in genetic diseases. Accordingly, the manuscript is organized around a variant‐mechanism‐driven editing strategy and translational‐readiness framework rather than as an encyclopaedic survey of CRISPR technologies. Literature was prioritized from PubMed, publisher pages, regulatory sources, and major clinical‐trial reports, with emphasis on primary mechanistic studies, pivotal translational studies, approved or advanced therapeutic programs, and recent reviews published up to June 2026. Studies were interpreted according to evidence level, separating approved therapies, active clinical trials, preclinical disease models, and conceptual technology development. Technologies primarily designed for transcript‐level modulation, circular RNA engineering, or transposase‐mediated integration are mentioned only where they clarify the boundaries of the genome‐editing framework.

## 2 MOLECULAR BASIS OF PATHOGENIC MUTATION SPECTRA AND GENE FUNCTIONAL IMBALANCE IN GENETIC DISEASES

### 2.1 Mutational features of monogenic genetic diseases, polygenic diseases, and complex genetic disorders

The mechanistic differences in genetic diseases first manifest in the distinct organizational patterns of pathogenic mutations.^7^ Monogenic diseases are typically dominated by a single high‐effect mutation, and the causal relationship between genotype and phenotype is relatively straightforward to establish; therefore, they are often used to dissect the direct connection among mutation, gene function, and disease. In contrast, polygenic genetic diseases and complex genetically associated disorders generally involve multiple moderate‐ to low‐effect variants and are jointly influenced by environmental exposure, developmental stage, tissue context, and genetic modifiers. This distinction dictates that different genetic diseases cannot be interpreted or edited using a single model. Some studies further suggest that the same variant may exhibit different penetrance and phenotypic severity across different genetic backgrounds or tissue environments, which is an important source of clinical heterogeneity in genetic diseases. Consequently, the genetic architecture not only determines the disease interpretation model but also influences the design of CRISPR‐based functional studies: mutations with high penetrance in single genes are more suitable for single‐point mutation reconstruction and isogenic background validation, whereas diseases involving low‐effect variants, modifier genes, and tissue context depend more on combinatorial perturbations, screening systems, and network‐level functional dissection.

From the perspective of gene function studies, the emphases of CRISPR applications differ across genetic disease types. For monogenic diseases, CRISPR studies often emphasize^6^, ^9^ the verification of pathogenic causality through mutation knock‐in, mutation correction, or allele‐specific regulation. For polygenic and complex genetically associated diseases, related studies tend to rely on CRISPR screening, combinatorial editing, and regulatory network analysis to identify key nodes, modifier genes, and pathway interactions. A synthesis of current research indicates that genetic diseases should not be simply understood as a linear process of ‘one mutation corresponding to one phenotype’ but rather as a pathological continuum shaped jointly by mutation effects, gene function, cellular state, and tissue microenvironment. Thus, the value of CRISPR in genetic diseases depends not only on whether a particular genetic locus can be altered but also on the functional hierarchy of that locus within the genetic architecture and disease network. This framework largely represents the organizational approach and trajectory of current research and does not imply that the fields of pathogenic mechanism dissection, tool delivery, safety evaluation, and clinical translation have reached equal maturity.

### 2.2 Pathogenic roles of point mutations, indels, copy number variations, and chromosomal structural variants

The structural type of pathogenic mutations directly determines their molecular consequences.^4^, ^10^ Point mutations can lead to missense mutations, nonsense mutations, splice‐site alterations, or disruption of regulatory elements; insertion–deletion variants may cause frameshifts, protein truncation, or loss of critical domains; copy number variations frequently disrupt pathway homeostasis by altering gene dosage. Studies^7^ indicate that certain chromosomal inversions, translocations, and complex rearrangements may also disrupt the three‐dimensional genome architecture, alter enhancer–promoter interactions, and even generate abnormal fusion genes. Evidence suggests that the impact of structural variants often extends beyond single‐gene boundaries; therefore, their functional interpretation requires integrated assessment of genomic location, regulatory domains, and tissue expression profiles.^11^

This structural diversity of variants also directly defines the boundaries for selecting CRISPR editing strategies.^6^ Previous studies indicate that single‐base substitutions are more amenable to editing modes capable of precise base rewriting; repair strategies for small insertions and deletions must be judged in light of cell‐division status, the feasibility of donor‐template delivery, and double‐strand break risk. For large deletions, duplications, inversions, and complex rearrangements, more sophisticated genome engineering strategies may be required. Therefore, classifying mutation types holds value not only for diagnosis but also for functional validation, disease model construction, and therapeutic editing design. Without precise characterization of the mutation structure, editing strategies may remain at a level of technical feasibility without truly addressing the disease mechanism.

### 2.3 Molecular mechanisms of loss‐of‐function, gain‐of‐function, and dominant‐negative mutations

Evidence further suggests^4^ that the structural form of DNA variants does not necessarily dictate their ultimate functional consequences; thus, mechanistic analysis of genetic diseases also requires distinguishing among loss‐of‐function mutations, gain‐of‐function mutations, and dominant‐negative mutations. Protein structure and function studies indicate that loss‐of‐function mutations typically cause insufficient pathway activity by reducing protein stability, disrupting catalytic sites, impairing folding, or blocking protein interactions. Gain‐of‐function mutations may confer abnormal activity on the protein, sustained signalling input, or new molecular interactions. Dominant‐negative mutations may interfere with normal allele function through abnormal protein products, causing a pathological impact that exceeds simple dosage reduction. Certain mutations may exhibit distinct functional consequences across different cell types or developmental stages.

Mechanistic studies usually emphasize this functional classification has direct guiding significance for precision editing. For loss‐of‐function diseases, precise repair, gene compensation, or expression restoration. For gain‐of‐function diseases, allele‐specific knockout, transcriptional repression, or disruption of abnormal functional domains may be more consistent with mechanistic logic in certain scenarios, but their feasibility still depends on mutant‐allele selectivity, gene‐dosage tolerance, and the requirement to preserve normal protein function. For dominant‐negative mutations, simply supplementing normal gene expression may not suffice, and it is also necessary to address the interference of the abnormal protein on normal complexes or signalling networks. Overall, ‘editability’ not only refers to whether a mutation can be rewritten but also whether the correct molecular functional direction can be restored after rewriting. Therefore, functional consequence analysis should be placed before the selection of editing strategies to avoid directly deriving intervention paths based solely on mutation forms.

### 2.4 Impact of coding and non‐coding region mutations on gene expression regulatory networks

Early genetic disease research focused more on coding region mutations because their effects on protein structure and function are relatively intuitive. With the development of whole‐genome sequencing, transcriptomics, and epigenomics, the pathogenic significance of non‐coding region variants has gradually gained attention. Genomic and regulatory studies suggest^5^, ^12^, ^13^, ^14^, ^15^ that variants in promoters, enhancers, UTRs, deep intronic regions, and splicing regulatory regions can alter gene expression by affecting transcriptional intensity, mRNA stability, splicing patterns, translation efficiency, and chromatin accessibility. Thus, variants that do not alter the protein sequence can still produce clear pathological consequences. This phenomenon illustrates that functional imbalance in genetic diseases can occur not only at the protein product level but also at the level of expression regulation and chromatin organization.

**FIGURE 2:**
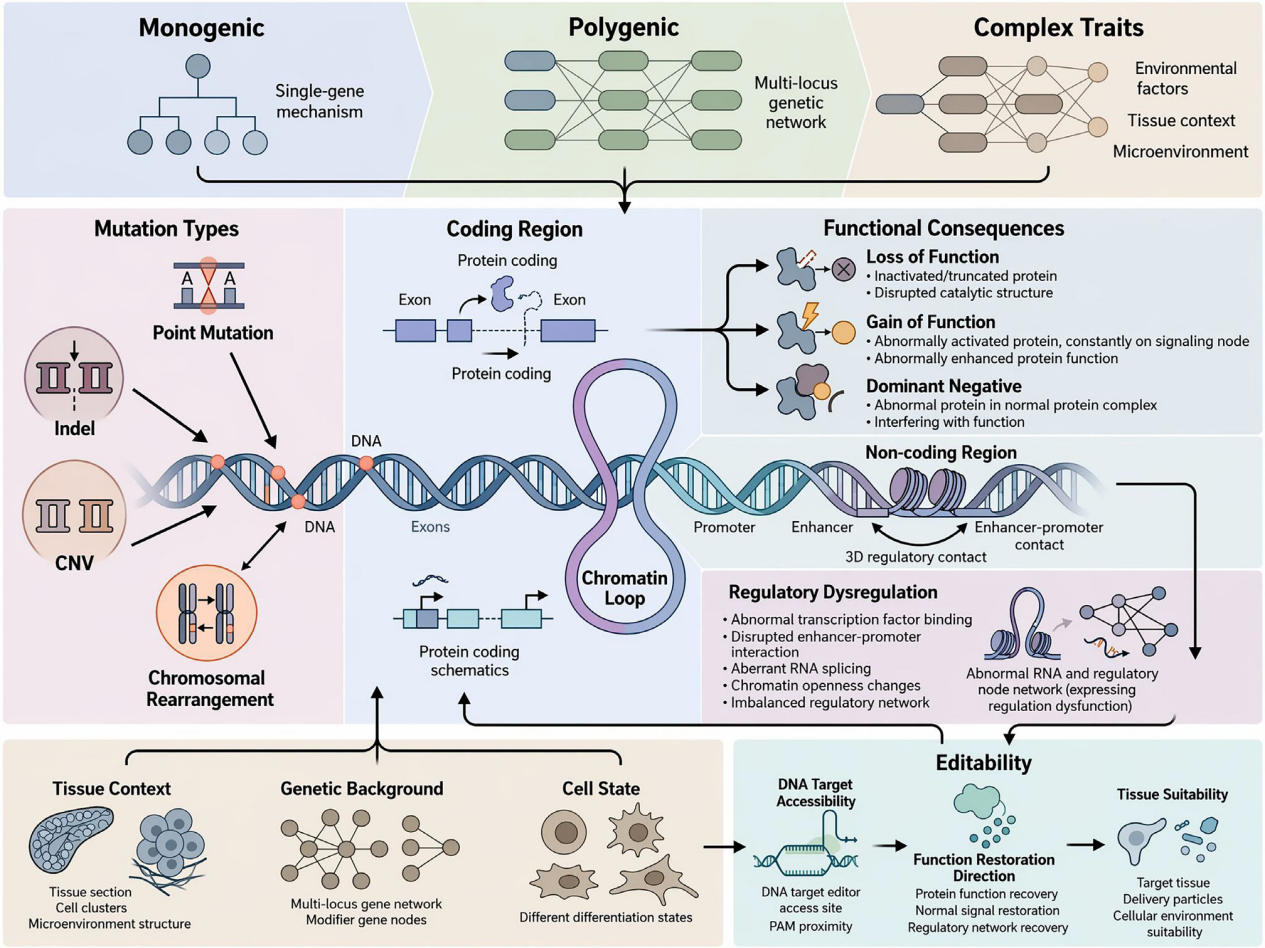
Logical relationships among pathogenic mutation spectra, functional consequences, and editability in genetic diseases.

Mechanistic interpretation of non‐coding region mutations often depends more on cell type, developmental stage, and chromatin context. Functional studies show that some non‐coding variants can disrupt transcription factor binding, enhancer–promoter interactions, or three‐dimensional genome architecture, thereby affecting key gene expression. For such variants, the value of CRISPR‐Cas9 lies not only in sequence repair but also in validating their functional consequences through CRISPRi, CRISPRa, epigenetic editing, or regulatory element deletion. Together^16^ non‐coding variant studies have expanded the mechanism of genetic diseases from ‘protein abnormality’ to ‘gene regulatory network imbalance’, and also provide a more complete framework for explaining complex phenotypes (Figure 2).

### 2.5 Correspondence among pathogenic mutation types, functional consequences, and CRISPR editability

Regarding precision editing strategies, a clear correspondence needs to be established among mutation structure, functional consequences, and editability. From a mechanistic classification perspective, the correspondence among mutation structure, functional consequences, and editability forms the basis for subsequent editing tool selection and repair strategy design. Current gene editing evidence indicates^6^, ^7^, ^17^ that the choice of editing strategy cannot be based solely on editing efficiency; it must also consider whether the mutation is in a targetable region, whether the editing product is predictable, whether the functional restoration direction is clear, and whether the target tissue has acceptable delivery conditions and a safety window. Therefore, editability in genetic diseases should be understood as a comprehensive concept spanning sequence, function, delivery, and safety, rather than simply target cleavability (Table 1).^18^, ^19^, ^20^, ^21^, ^22^

**TABLE 1:** Matrix linking pathogenic variant classes, functional consequences, and CRISPR strategy selection in genetic diseases

Variant class	Molecular consequence	Disease/gene context	CRISPR strategy selection	Evaluation metrics	Key limitations
Missense SNV^23^, ^24^	Amino‐acid substitution; altered folding, activity, or domain function	Monogenic pathogenic SNVs	Assess direct correction by base editing or prime editing; validate mutant and corrected models	Correction rate; bystander editing; protein activity; phenotype rescue	PAM/editing‐window constraints; bystander bases; delivery efficiency
Nonsense SNV^24^	Premature stop codon; truncated protein; nonsense‐mediated decay	Loss‐of‐function monogenic diseases	Use base editing when editable; consider prime editing or readthrough only when mechanism supports it	Stop‐codon correction; mRNA stability; full‐length protein; functional recovery	Editable windows are limited; sequence repair may not restore splicing, translation, or folding
Small indel^18^, ^25^	Frameshift, truncation, or domain loss	CFTR, metabolic enzymes, structural proteins	Choose HDR or prime editing according to indel length, cell‐cycle state, and donor feasibility	Indel correction; edited‐product purity; protein restoration; functional direction	Prime‐editing efficiency is locus dependent; HDR requires donor delivery and dividing cells
Splice‐site/deep‐intronic variant^5^, ^14^	Exon skipping, cryptic splice activation, or abnormal transcript	RNU4‐2 and other splicing disorders	Define splicing mechanism before selecting base editing, prime editing, CRISPRi/a, or regulatory editing	Normal‐splicing fraction; abnormal transcript burden; protein recovery	Splicing is cell‐type and developmental‐stage dependent
Non‐coding regulatory variant^13^, ^19^	Altered promoter/enhancer activity, transcription‐factor binding, or chromatin accessibility	Cell‐type‐specific enhancers, promoters, cis‐regulatory elements	Use CRISPRi/a, epigenome editing, or perturbation before considering precise repair	Expression level; enhancer activity; chromatin accessibility; 3D contacts	Highly context‐dependent; functional direction may be hard to predict
Copy‐number variant^20^	Dosage imbalance and pathway disturbance	Deletion/duplication syndromes; dosage‐sensitive genes	Prioritize dosage‐effect studies; consider CRISPRa/i or compensation when biologically justified	Gene dosage; pathway activity; cell state; tissue phenotype	Large dosage defects are difficult to resolve by single‐locus editing
Structural variant/3D genome disruption^21^, ^22^	Inversion, translocation, enhancer hijacking, topological disruption, or fusion gene	CTCF‐related 3D genome abnormalities and developmental disorders	Use CRISPR models to clarify mechanisms; evaluate large‐fragment engineering cautiously	3D chromatin interaction; expression; fusion product; developmental phenotype	Repair may generate structural by‐products and genome‐stability risks

*Note*: Superscript numbers indicate representative references for the corresponding category.

Therefore, CRISPR strategy design in genetic diseases should shift from ‘disease name orientation’ to ‘variant mechanism orientation’.^6^, ^17^ For example, the evaluation of strategies for nonsense mutations focuses on the ability to restore effective coding sequences, modulate aberrant transcripts, or alleviate premature termination effects. The selection of strategies for splicing abnormalities requires consideration of mutation location, splicing regulatory context, and target cell types; whether gain‐of‐function or dominant‐negative mutations are suitable for selective silencing of aberrant alleles also necessitates evaluation of allele specificity, gene dosage tolerance, and the requirement for normal function preservation. Based on current research, editability encompasses both whether the target sequence can be recognized by the CRISPR system and whether editing can stably restore gene function, cellular state, and tissue phenotype. This conceptual framework shifts editing strategies for genetic diseases from disease‐name‐driven to variant‐mechanism‐driven, and provides a clearer logical basis for functional studies and mutation repair.

## 3 CRISPR‐Cas9 EDITING SYSTEMS AND THEIR APPLICATION IN GENE FUNCTION STUDIES FOR GENETIC DISEASES

### 3.1 Composition, targeting recognition mechanisms, and editing outcomes of CRISPR‐Cas9 systems

The key value of CRISPR‐Cas9 lies not solely in its nuclease activity, but in its redirection to different genomic loci via sgRNA, enabling the reconstruction, correction, or modulation of specific pathogenic mutations within the endogenous chromatin context in genetic disease research.^7^, ^17^, ^26^ Compared with early editing tools such as zinc finger nucleases and TALENs, methodological studies suggest that CRISPR‐Cas9 achieves target redirection primarily by altering the sgRNA sequence, thereby reducing the complexity of protein engineering. For genetic disease research, this programmability not only enhances target design efficiency, but more importantly, allows pathogenic loci, candidate genes, and regulatory regions to be systematically incorporated into a functional validation framework. By comparing mutant, corrected, and isogenic background control models, CRISPR‐Cas9 can help minimize interpretational biases introduced by exogenous expression or random mutation models.

Regarding editing outcomes, CRISPR‐Cas9‐mediated DNA double‐strand breaks do not automatically yield a single result; instead, they are determined by intracellular DNA repair pathways. Studies indicate that^27^ double‐strand breaks can initiate repair processes such as non‐homologous end joining, homology‐directed repair, or alternative end joining. Non‐homologous end joining often produces small insertions or deletions and is thus frequently used to construct gene knockout models; homology‐directed repair, with the participation of donor templates, can achieve specific sequence knock‐in or mutation correction. Based on current research, the value of CRISPR‐Cas9 in genetic diseases is not merely to alter DNA sequences, but to provide a technical foundation for establishing the causal chain of ‘endogenous locus alteration—gene function change—disease phenotype formation’.

### 3.2 Non‐homologous end joining, homology‐directed repair, and their roles in gene knockout, knock‐in, and mutation repair

Different DNA repair pathways endow CRISPR‐Cas9 with distinct utility in functional research.^7^, ^17^ Methodological studies indicate that NHEJ is relatively active in various mammalian cells, and its repair process does not rely on homologous templates; thus, it readily generates editing products such as small insertions or deletions. For loss‐of‐function studies, this imprecision offers practical value because frameshift mutations, premature stop codons, or the disruption of critical domains can inactivate the target gene. In previous functional studies of genetic diseases, NHEJ‐mediated gene knockout strategies have often been used to assess associations between the loss of candidate genes and specific cellular phenotypes, disease pathways, or tissue functional abnormalities.

In contrast to NHEJ, HDR is more suitable for precise knock‐in, pathogenic mutation modelling, and sequence correction.^28^ Technical studies indicate that HDR depends on donor templates, cell cycle status, and repair pathway activity; therefore, it is more likely to yield desired editing products in actively dividing cells, whereas its efficiency is typically restricted in terminally differentiated cells or in vivo tissues. To address this limitation, research has proposed methods such as donor template optimization, cell cycle regulation, DNA repair pathway modulation, and delivery timing optimization. Taken together, current research suggests that neither NHEJ nor HDR is universally superior: the former is more suitable for disrupting gene function, while the latter is more appropriate for modelling or correcting specific pathogenic mutations (Figure S1).

### 3.3 CRISPR‐based gene knockout, knock‐in, conditional editing, and transcriptional regulation strategies

Functional studies of genetic diseases require distinguishing between two levels: whether a gene is essential and how a specific mutation alters function. Studies^9^ indicate that CRISPR‐based gene knockout is suitable for verifying whether the loss of a candidate gene is sufficient to induce disease‐related phenotypes, especially for loss‐of‐function mutations or the screening of disease‐protective genes. In contrast, knock‐in strategies are better suited for modelling patient‐specific mutations at endogenous loci, thereby reducing dosage biases introduced by overexpression systems. For dominant mutations, regulatory mutations, or subtle functional alterations, studies suggest that simple gene knockout may not recapitulate the true pathological state, and mutation knock‐in and allele‐specific editing often provide greater mechanistic explanatory value.

Beyond DNA sequence editing, CRISPR‐derived transcriptional regulation systems have expanded the dimensions of mechanistic studies on genetic diseases. Studies report that the fusion of catalytically inactive Cas9 with transcriptional repressor or activator domains can create CRISPRi and CRISPRa systems, which downregulate or enhance target gene expression without altering the DNA sequence. Such strategies are highly suitable for analyzing dosage‐sensitive genes, non‐coding regulatory elements, and developmental stage‐specific expression abnormalities. For certain genetic diseases driven by expression imbalance, CRISPRi/a can help distinguish the effects of sequence alterations from those of expression level changes. Consequently, CRISPR‐based functional research has evolved from simple gene knockout to a multi‐layered system encompassing gene knockout, knock‐in, transcriptional regulation, and epigenetic modulation.

### 3.4 Target gene knockout in cellular and animal models for validating gene‐specific roles

CRISPR‐mediated target gene knockout remains a necessary specificity test when assigning pathogenic roles to candidate genes. In cellular systems, knockout can determine whether loss of a target gene is sufficient to alter disease‐relevant molecular pathways, cell viability, differentiation, or functional phenotypes; in animal models, tissue‐ or organism‐level knockout can further test whether the same perturbation produces systemic phenotypes consistent with the genetic disease mechanism. However, knockout should be interpreted as a functional validation strategy rather than as a direct therapeutic surrogate. Robust inference usually requires rescue experiments, correction of the mutant allele, or isogenic controls to distinguish target‐specific effects from off‐target editing, clonal bias, compensatory pathway activation, and non‐specific stress responses.

### 3.5 Application of CRISPR screening technology in the identification of causative genes for genetic diseases

Mechanistic studies of genetic diseases often face challenges such as a large number of candidate genes, high phenotypic heterogeneity, and complex pathway interactions. Methodological studies indicate that CRISPR screening can systematically assess the relationships between gene perturbations and cell survival, differentiation states, signalling activities, or disease‐related phenotypes using genome‐wide or focused single‐guide RNA libraries. In contrast to traditional candidate gene validation, evidence suggests that CRISPR screening is more suitable for unbiased discovery of disease dependency genes, phenotypic modifiers, and potential therapeutic targets. For polygenic genetic diseases and complex genetically associated disorders, this strategy is particularly helpful in identifying functional network nodes that are difficult to explain by single variants.

With the development of single‐cell omics and high‐content phenotyping technologies, the readout of CRISPR screening has shifted from simple enrichment or depletion analysis to perturbation‐omics frameworks.^9^, ^29^, ^30^ Available evidence indicates that^31^ methods such as Perturb‐seq can combine CRISPR perturbations with single‐cell transcriptomic readouts to dissect how specific genetic alterations affect cell states, differentiation trajectories, and regulatory networks. Related studies at the cellular level suggest that these approaches can reveal functional dependency relationships and pathway hierarchy effects between genes. Taken together, current research indicates that the value of CRISPR screening lies not only in discovering candidate genes but also in translating genetic variants into interpretable functional network maps.

### 3.6 CRISPR‐mediated functional validation strategies: from candidate variants to causal relationship confirmation

The key to high‐level genetic disease research is not merely discovering candidate variants but demonstrating the causal relationship between the variant and disease‐related phenotypes through reproducible and controlled functional perturbations. In the CRISPR‐mediated functional validation pipeline, candidate pathogenic genes can be assessed for their functional necessity by gene knockout, for whether specific sites are sufficient to induce disease‐related phenotypes by mutation knock‐in, for the reversibility of abnormal phenotypes by mutation correction or rescue experiments, and for whether upregulation or downregulation of gene expression is sufficient to alter disease‐related cellular states by CRISPRi/a analysis. Compared with simple correlation analysis, this multi‐directional validation strategy of ‘knockout—knock‐in—correction—rescue—expression regulation’ is more conducive to establishing a causal evidence chain of ‘candidate variant—gene function alteration—cellular state abnormality—disease‐related phenotype.’ Therefore, this section emphasizes the technical path of CRISPR in causal relationship confirmation rather than any specific model system itself; the specific model types and their applicable boundaries will be further elaborated in subsequent sections.

When interpreting CRISPR functional validation results, editing efficiency should not be directly equated with mechanistic proof. Methodological reviews point out that editing product heterogeneity, clonal selection bias, off‐target effects, cellular adaptive responses, and model system differences can all affect the reliability of conclusions. Therefore, CRISPR functional validation often requires the combination of multiple sgRNA designs, independent clonal verification, mutation rescue, orthogonal perturbation methods, and multi‐level phenotypic readouts to reduce interpretational bias caused by a single editing event or a single readout. Taken together, current research indicates that the core contribution of CRISPR‐Cas9 is not simply improving the efficiency of gene manipulation, but enabling the functional necessity, pathogenic sufficiency, and phenotypic reversibility of candidate variants to be continuously evaluated within the same validation framework.

## 4 CRISPR‐BASED MODEL DISSECTION OF GENETIC DISEASE MECHANISMS: FROM CELL MODELS TO ORGANOID SYSTEMS

### 4.1 Application of CRISPR‐edited cell models in functional validation of pathogenic mutations

After establishing the overall framework for CRISPR‐based functional validation strategies, cell models provide the most fundamental and controllable experimental system for studying pathogenic mutations. By introducing patient‐derived mutations, knocking out candidate genes, or correcting abnormal sequences in identical or highly similar genetic backgrounds, CRISPR‐Cas9 can be used to construct gene‐edited cell models, including mutant, corrected, and wild‐type lines, thereby enabling comparison of molecular functional differences across different genetic states. Compared with exogenous overexpression systems, isogenic endogenous editing models more closely recapitulate authentic gene dosage and chromatin environment, reducing interference from genetic background differences in phenotypic interpretation, and are therefore more suitable for analyzing the direct impact of specific mutations on protein function, signalling pathways, and cellular phenotypes.

Isogenic cell models are better suited for dissecting the direct molecular consequences of single mutations, single gene perturbations, or specific regulatory element alterations, but their capacity to simulate tissue architecture, cell–cell interactions, and long‐term disease progression is limited.^32^, ^33^, ^34^ Meanwhile, the genetic background, chromatin accessibility, and DNA repair capacity of different cell lines may still affect editing outcomes and phenotypic interpretation. For genetic diseases with strong tissue specificity, developmental stage dependency, or involvement of multi‐cellular interactions, conventional cell models often fail to fully recapitulate the authentic pathological environment. Therefore, isogenic cell models should be positioned as a starting point for functional validation of pathogenic variants and a platform for molecular mechanism dissection, rather than as a complete surrogate system for complex disease phenotypes.

### 4.2 Induced pluripotent stem cell models recapitulate patient‐specific genetic background and developmental abnormalities

The pathological basis of many genetic diseases is not confined to mature tissues but emerges progressively during cell differentiation, tissue formation, and developmental maturation. Studies indicate that^35^ patient‐derived iPSCs retain individual genetic backgrounds and can be differentiated into disease‐relevant cell types such as neurons, cardiomyocytes, hepatocytes, and blood cells, thus enabling analysis of the functional consequences of patient‐specific mutations along developmental trajectories. CRISPR‐Cas9 further enhances the causal validation capacity of iPSC models, and evidence suggests that mutation correction and mutation knock‐in can be used to establish isogenic background controls.

The value of iPSC models lies in bridging human genetic backgrounds with manipulable differentiation systems, yet their interpretive scope remains limited.^35^ Studies indicate that the reprogramming process may alter epigenetic states, and the maturity of differentiated products may be lower than that of adult tissues, which can affect mechanistic interpretation of late‐onset or age‐related genetic diseases. For neurodegenerative diseases, cardiomyopathies, and metabolic genetic disorders, related studies often enhance model relevance through long‐term culture, three‐dimensional culture, stress conditions, or tissue‐like systems. Taken together, the significance of combining CRISPR with iPSCs lies in constructing a causal evidence chain supported collectively by isogenic backgrounds, developmental trajectories, and disease‐relevant phenotypes.

### 4.3 Organoids unravel tissue‐specific gene function and disease phenotypes

When genetic diseases involve complex tissue architecture, intercellular interactions, or spatial developmental programs, two‐dimensional cell models typically offer limited explanatory power. Studies indicate that systems such as brain organoids, retinal organoids, intestinal organoids, kidney organoids, and liver organoids can, to some extent, simulate tissue‐specific cell composition, developmental hierarchy, and local microenvironment. Different organoid systems correspond to distinct mechanistic scenarios of genetic diseases; for instance, brain and retinal organoids are more suitable for dissecting neurodevelopmental abnormalities or photoreceptor‐related genetic disorders, whereas kidney and liver organoids are better suited for exploring tissue‐specific metabolic, structural, and developmental abnormalities. Combined with CRISPR‐Cas9, evidence suggests that organoid models can be employed to analyze how specific mutations affect cell lineage differentiation, tissue structure formation, and regional functional maturation.

Evidence also shows^12^ that the mechanistic value of organoid models is particularly evident in genetic diseases that are strongly dependent on developmental windows. Related studies have observed that certain mutations may manifest only limited molecular abnormalities in conventional cell models but can be interpreted as altered cell proportions, disrupted tissue architecture, or shifted developmental programs in three‐dimensional organoid systems. However, organoids still exhibit limitations such as insufficient maturity, batch variability, lack of vascularization, and inadequate immune components. Taken together, current research indicates that CRISPR‐organoid models are better suited to address the mechanistic question of how mutations disrupt tissue formation and cell–cell interactions, rather than to fully replace in vivo disease models.

### 4.4 Role of animal models in verifying pathogenesis and therapeutic strategies for genetic diseases

Genetic diseases ultimately often manifest as organ dysfunction and clinical phenotypes at the organismal level; therefore, animal models remain a key component in mechanistic validation and therapeutic strategy evaluation. Studies indicate that^36^, ^37^ CRISPR‐Cas9 has improved the efficiency of constructing pathogenic mutation models in mice, zebrafish, rats, and other model organisms, making gene knockout, point mutation knock‐in, and conditional editing models more readily applicable to genetic disease research. Compared with cell and organoid models, evidence suggests that^38^ animal models can recapitulate intertissue interactions, immune responses, metabolic states, and long‐term disease progression, making them suitable for analyzing the impact of genetic mutations on the entire physiological system.

The translational value of animal models must be understood alongside their limitations. Evidence suggests that^32^, ^39^ certain human pathogenic mutations may not produce fully consistent disease phenotypes in mice or other model animals, potentially due to differences in gene redundancy, developmental speed, tissue architecture, and compensatory mechanisms. For non‐coding regulatory variants or human‐specific developmental processes, the explanatory power of animal models must be evaluated in conjunction with evidence from human cells, iPSCs, and organoids. Collectively, current research suggests that animal models should not be regarded as a single ultimate proof but rather as a key link in a multi‐model evidence chain that connects molecular mechanisms, tissue pathology, and therapeutic feasibility (Table 2).^40^, ^41^

**TABLE 2:** Comparative applicability, advantages, and limitations of CRISPR‐based genetic disease model systems

Model system	Primary application	Mechanistic level	Core advantages	Major limitations	Recommended validation
CRISPR‐edited cellular model^9^, ^39^	Test whether a mutation, gene, or regulatory locus has a direct functional effect	DNA, RNA, protein, cellular phenotype	Highly controllable; supports mutant, corrected, and isogenic controls	Limited tissue architecture, long‐term progression, and microenvironment	Combine with iPSC, organoid, or animal models for tissue‐level validation
CRISPR‐edited iPSC/isogenic iPSC model^40^, ^42^	Analyze patient‐background and developmental effects	Genetic background, differentiation stage, cell fate	Preserves patient background; correction/knock‐in reduces background variation	Reprogramming, immature differentiation, and batch effects	Validate against primary cells, organoids, or animal models
CRISPR organoid/assembloid model^9^, ^39^	Define effects on tissue formation, lineage differentiation, and cell‐cell interaction	3D architecture, spatial programs, cellular composition	Models tissue‐specific composition and development	Limited maturation, vascularization, immune components, and reproducibility	Combine with single‐cell/spatial omics and animal validation
Organoid CRISPR screening model^9^, ^41^	Identify disease genes, modifiers, or drug‐sensitivity nodes in 3D context	Perturbation, cell‐state change, pathway dependency	Supports knockout, CRISPRi, CRISPRa, and single‐cell readouts	Library coverage, transduction efficiency, heterogeneity, analysis complexity	Cross‐check with 2D screens and focused functional validation
CRISPR animal model^36^, ^37^	Assess organ function, systemic phenotype, immune response, and long‐term course	Organismal phenotype and organ crosstalk	Captures systemic pathology and long‐term progression	Species differences, redundancy, and compensation	Use with human cells/organoids to avoid overinterpreting animal‐only evidence
In vivo therapeutic‐validation animal model^38^	Evaluate delivery, target‐tissue editing, efficacy, and safety	Tissue targeting, expression duration, safety, functional benefit	Tests editing efficiency, distribution, benefit, and toxicity together	Human translation is limited by species and immune differences	Combine with human models and long‐term safety assays
Integrated multi‐model framework^17^, ^37^, ^39^	Build evidence from variant to cell, tissue, and organism	Molecular‐cellular‐tissue‐organismal chain	Reduces single‐model bias; validates rescue and feasibility across levels	Costly and time‐intensive; endpoints may differ across models	Use staged validation: cellular mechanism → iPSC/organoid tissue context → animal/systemic phenotype

*Note*: Superscript numbers indicate representative references for the corresponding category.

### 4.5 Advantages, limitations, and mechanistic research value of CRISPR model systems

From a holistic research perspective, the primary contribution of CRISPR models lies in advancing genetic disease studies from correlational associations to verifiable causal analyses. Studies indicate that^17^, ^35^, ^36^, ^39^ different CRISPR models are not mutually replaceable but correspond to distinct levels in the mechanistic chain of genetic diseases. Regarding model selection, cell models are more suited for elucidating direct molecular consequences, iPSC models are better for preserving patient genetic backgrounds and developmental trajectories, organoid models are more appropriate for investigating tissue organization and cell–cell interactions, and animal models are better for evaluating systemic pathology and long‐term effects. The value of combining multiple models lies in reducing bias from any single model rather than simply increasing experimental complexity; therefore, rational model selection should depend on disease characteristics, mutation types, and the specific scientific question.

**FIGURE 3:**
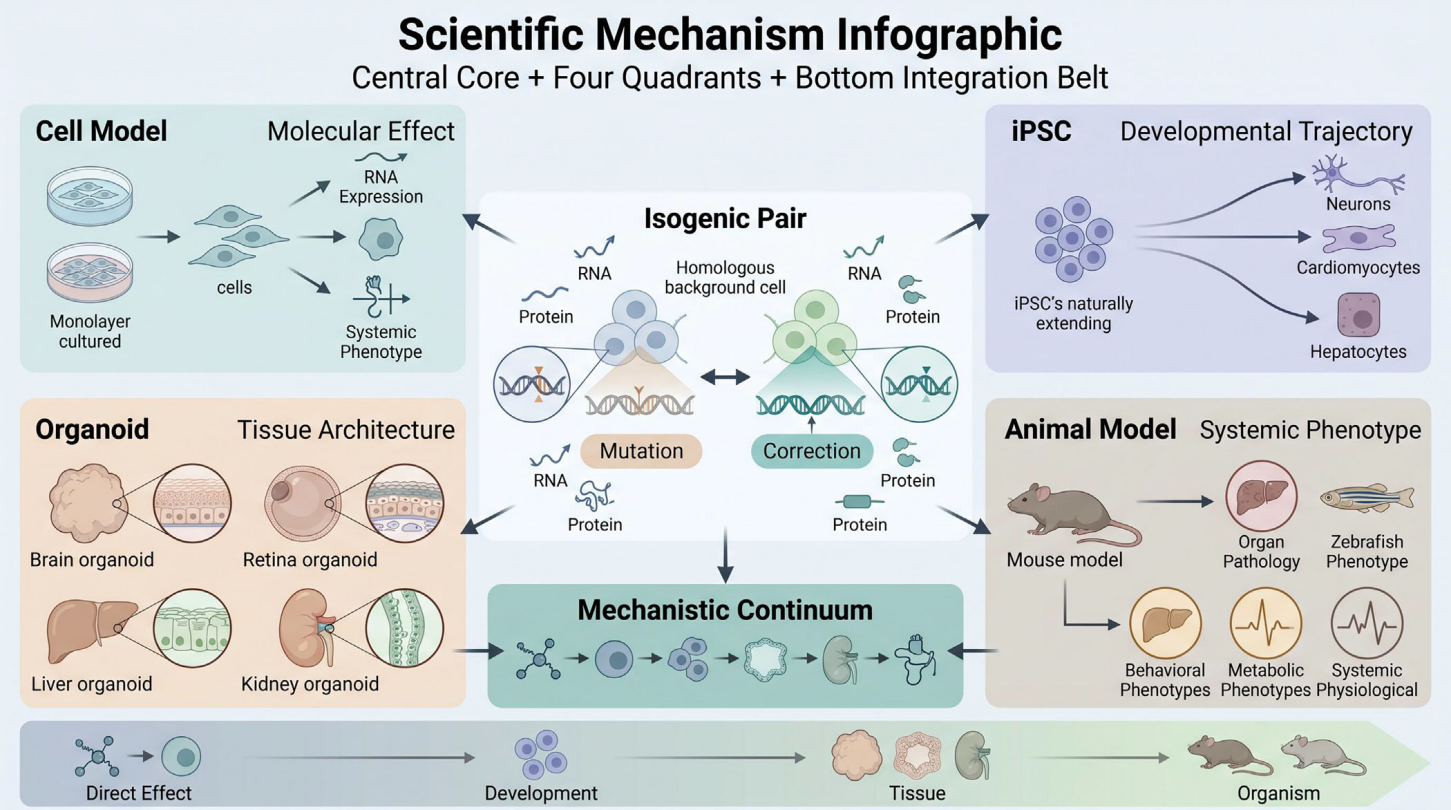
Positioning of CRISPR multi‐layer disease models in elucidating mechanisms of genetic diseases.

The limitations of these model systems also represent challenges that mechanistic studies must address. Methodological studies suggest^26^, ^35^, ^39^, ^43^ that CRISPR editing may introduce clonal bias, editing product heterogeneity, off‐target effects, or altered cellular fitness; iPSCs and organoids can be affected by maturity, batch variability, and culture conditions; and animal models may be constrained by species differences. Consequently, robust mechanistic interpretation generally requires support from isogenic controls, mutation rescue experiments, multi‐model validation, and multi‐level phenotypic assessment. Taken together, current research demonstrates that the value of the CRISPR model system lies not only in constructing disease models but in establishing a continuous mechanistic framework from pathogenic variants to cellular function, tissue architecture, and organismal phenotype (Figure 3).

## 5 CRISPR‐DERIVED PRECISION EDITING TOOLS EXPAND THE BOUNDARIES OF MUTATION REPAIR IN GENETIC DISEASES

### 5.1 Advantages and limitations of classical CRISPR‐Cas9 double‐strand break editing

Classical CRISPR‐Cas9 double‐strand break editing has provided a foundation for constructing genetic disease models and early mutation repair; however, its therapeutic application is constrained by risks associated with double‐strand breaks, limited HDR efficiency, and editing product heterogeneity.^6^, ^7^, ^23^, ^44^, ^45^ In gene function studies of genetic diseases, classical CRISPR‐Cas9 remains valuable, particularly for gene knockout, mutation simulation, large‐fragment engineering, and certain sequence repair scenarios that depend on donor templates. Related studies indicate that this system can help establish functional links between pathogenic genes and disease phenotypes and provide mechanistic references for subsequent precision repair strategies. Nevertheless, the limitations of the classical Cas9 system have driven the development of more refined and less damaging editing tools, gradually shifting mutation repair in genetic diseases from double‐strand break‐dependent editing towards mechanism‐adapted precision rewriting.

The application value of double‐strand break editing must be understood together with its potential risks. Methodological studies indicate^6^, ^43^, ^44^ that Cas9‐induced double‐strand breaks may be accompanied by editing product heterogeneity, large deletions, chromosomal rearrangements, DNA damage responses, and cellular selection pressure. Related studies further suggest that HDR efficiency is influenced by cell cycle status, cell type, and delivery conditions and is often limited in terminally differentiated cells or in vivo tissues. For genetic diseases caused solely by single‐nucleotide changes, inducing double‐strand breaks may exceed the actual repair requirement. Collectively, current research indicates that classical CRISPR‐Cas9 remains an important foundational tool for mechanistic studies but requires complementation with less damaging, higher‐precision editing strategies for therapeutic mutation repair.

For therapeutic development, canonical double‐strand break editing also requires explicit genotoxicity assessment beyond conventional indel quantification. On‐target cleavage can generate large deletions extending far beyond the intended edit, megabase‐scale chromosomal truncations, chromosomal translocations, and complex rearrangement patterns such as chromothripsis, particularly in proliferative stem or progenitor cells that may undergo expansion after editing. These risks are mechanistically distinct from simple off‐target cleavage because they can arise at the intended locus and may persist in clonally expanded products. Therefore, DSB‐dependent strategies should be reserved for contexts in which knockout or large‐fragment engineering is mechanistically justified, and they should be complemented by long‐read sequencing, structural variant analysis, karyotyping, and clonal expansion assessment when considered for therapeutic use.

### 5.2 Progress in base editing for precise correction of single‐nucleotide mutations

Single‐nucleotide substitutions represent a common type of pathogenic mutation in genetic diseases; therefore, base editing has become an important technical direction in precision editing. Technical studies indicate^17^ that base editing^23^, ^24^, ^46^ is typically formed by fusing a catalytically impaired or nicking Cas protein with a deaminase, enabling specific base conversions without generating classical double‐strand breaks or relying on donor templates. Existing studies suggest that cytosine base editors mainly mediate C‐to‐T conversions, while adenine base editors mainly mediate A‐to‐G conversions, making them suitable for correcting certain missense mutations, nonsense mutations, and splicing‐related variants. Moreover, the emergence of novel tools such as C‐to‐G base editing, glycosylase‐related base editing, dual base editing, and mitochondria‐related base editing suggests that the base editing lineage is still expanding; however, their disease compatibility, editing windows, and long‐term safety still require systematic evaluation. For monogenic diseases with well‐defined pathogenic mechanisms, available evidence supports base editing^46^, ^47^ as an important tool connecting point mutation identification and precision repair.

The applicability of base editing is still constrained by multiple factors. Studies indicate that the^48^ editing window, PAM location, convertible base types, and the distribution of identical bases within the target region all affect the final editing outcome. If multiple identical editable bases exist near the target site, bystander editing may lead to additional functional consequences. Related studies also suggest that deaminase activity may cause unintended alterations at the DNA or RNA level, so safety evaluation must not focus solely on whether the target base is corrected. Based on current research, base editing is more suitable for genetic disease mutations with a clearly defined target base, low bystander risk, and a well‐understood direction of functional recovery after editing, and it should not be regarded as a universal solution for all point mutations.

### 5.3 Prime editing potential for repairing small insertions, deletions, and complex mutations

For mutation types that cannot be covered by base editing, prime editing provides a more flexible approach for sequence rewriting. Technical studies indicate^7^ that prime editing typically consists of a nicking Cas9 fused to a reverse transcriptase, and uses a pegRNA to provide both the targeting sequence and the editing template, thus enabling base substitutions, small insertions, and small deletions without generating classical double‐strand breaks. Compared with base editing, existing studies suggest that prime editing^16^ can theoretically cover a wider range of genetic disease variants, and is particularly suitable for non‐C‐to‐T or non‐A‐to‐G conversions, small indels, and certain splicing abnormalities. This technique is therefore regarded as an important candidate strategy for the precision repair of complex small variants.

The translational potential of prime editing still requires comprehensive evaluation in combination with efficiency, product purity, and delivery conditions. Methodological studies show^45^, ^49^, ^50^ that prime editing^49^, ^50^, ^51^ efficiency is influenced by pegRNA structure, reverse transcription template length, target site sequence context, cell type, and DNA repair background, with significant variation among different sites. Related studies also suggest that the relatively complex composition of this system may increase the difficulty of in vivo delivery, especially when AAV capacity is limited or tissue‐specific expression control is required. Although prime editing safety assessments still require^26^ to reduce the risks associated with double‐strand breaks, the need to evaluate unintended by‐products, editing heterogeneity, and long‐term stability. Based on current research, prime editing^26^ expands the boundaries of mutation repair in genetic diseases, but its clinical feasibility still depends on the simultaneous optimization of editing efficiency, delivery systems, and safety evaluation.

### 5.4 Cas protein variants and PAM‐restriction engineering: enhancing targeting range and editing flexibility

Whether CRISPR editing can act on a specific pathogenic mutation often depends on the presence of a suitable PAM sequence near the target site. Comparative studies report^27^, ^52^, ^53^, ^54^ that classical SpCas9 has specific PAM requirements, which limits the editability of certain genetic disease loci. To address this bottleneck, various Cas protein variants and engineered Cas systems have been developed to broaden PAM recognition, improve editing flexibility, or enhance targeting specificity. Technical studies indicate that Cas9‐NG, SpG, SpRY, and certain Cas12a systems can recognize a wider range of PAM sequences or produce different DNA cleavage characteristics, thereby increasing the potential target selection space.

The significance of Cas protein variants lies not only in expanding the editable region but also in improving the compatibility between editing tools and specific mutations. For certain point mutations, the ideal editing window must precisely cover the target base; if the protospacer adjacent motif (PAM) position is unsuitable, even if base editing or prime editing^26^ possesses theoretical repair capacity, effective rewriting may be difficult to achieve. Evidence suggests that overcoming PAM constraints can be combined with base editing, prime editing, and transcriptional regulation systems to increase the design freedom of editing strategies for genetic diseases. Meanwhile, available evidence also indicates that different Cas protein variants differ in activity, specificity, molecular size, immunogenicity, and delivery compatibility; therefore, they cannot be evaluated solely by PAM range. Overall, Cas protein engineering is increasing the target selection freedom of CRISPR systems and providing designable editing windows for more genetic disease variants.

In addition to Cas9 and Cas12a systems, CRISPR‐Cas3 has emerging relevance for contexts in which programmed long‐range DNA degradation or targeted deletions are mechanistically useful. Recent disease‐model studies suggest that Cas3‐based editing may support large targeted deletions, such as in transthyretin amyloidosis models, but its broad therapeutic applicability remains constrained by deletion‐size control, delivery, and safety evaluation. From the perspective of tool adaptation, base editing emphasizes the precision of single‐base conversion, prime editing emphasizes the flexibility of rewriting small‐fragment variants, and Cas protein variant engineering primarily addresses target accessibility and editing window constraints; these tools do not constitute a linear replacement relationship but jointly serve strategy selection determined by mutation type, repair goal, and site conditions (Table 3).^55^, ^56^

**TABLE 3:** Comparative repair capacity, applicability boundaries, and safety limitations of CRISPR‐derived precision editing tools

Editing tool	Primary mode	Suitable variants/applications	Core advantages	Major limitations	Safety concerns
Canonical CRISPR‐Cas9 DSB editing^43^, ^44^, ^57^	sgRNA‐guided DSB followed by NHEJ, HDR, or alternative repair	Knockout, mutation modelling, large‐fragment engineering, selected HDR repair	Mature; useful for mechanistic studies and disease‐model construction	HDR depends on cell cycle, donor template, and delivery; low‐precision repair in non‐dividing tissues	Large deletions, complex rearrangements, chromothripsis, chromosomal truncation, DNA‐damage response, and heterogeneous products
Base editing^23^, ^24^	Cas nickase or catalytically impaired Cas fused to deaminase	Point mutations, selected stop codons, selected splice variants	Donor‐free single‐base correction without canonical DSBs	Restricted by PAM, editing window, target base, and local sequence	Guide‐dependent bystander editing within the editing window; guide‐dependent DNA off‐target editing; guide‐independent transcriptome‐wide RNA off‐targeting; deaminase‐related unintended edits
Mitochondrial base editing^55^	Mitochondria‐targeted base editors rewrite selected mtDNA sites	mtDNA point mutations, mitochondrial disease modelling	Bypasses conventional CRISPR delivery limits in mitochondria	Constrained by mitochondrial delivery, heteroplasmy, target context, and editing window	mtDNA bystander editing, heteroplasmy shift, and long‐term mitochondrial functional risk
Prime editing^14^, ^25^	Cas9 nickase‐reverse transcriptase plus pegRNA‐directed local rewriting	Non‐C‐to‐T/non‐A‐to‐G conversions, small indels, selected complex variants	Enables flexible small edits without DSBs or donor DNA	pegRNA design is complex; efficiency is locus‐dependent; system is large for in vivo delivery	pegRNA‐associated by‐products, product heterogeneity, nicking‐related genotoxicity, long‐term stability, and delivery‐associated risks
Cas variants/expanded PAM systems^52^, ^53^, ^54^	Engineered Cas proteins alter PAM recognition, specificity, or size	PAM‐constrained loci and poorly covered pathogenic variants	Expands editable loci; can be paired with base editing, prime editing, or regulation tools	Activity, specificity, size, and delivery compatibility vary substantially	Variant‐specific off‐target profiles, immunogenicity, and efficiency‐specificity trade‐offs
CRISPRi/a transcriptional regulation^16^, ^19^	dCas fusion proteins repress or activate gene/regulatory‐element activity	Dosage‐sensitive genes, regulatory elements, expression‐imbalance disorders	Reversible and tunable; useful for non‐coding mechanism studies	Does not repair DNA; depends on chromatin state, cell type, and expression background	Persistent expression, off‐target regulation, and over‐ or under‐correction of dosage
CRISPR epigenome editing^56^, ^58^	dCas‐linked methylation, demethylation, or histone‐modifying modules	Regulatory abnormalities and gene‐expression control without sequence change	Modifies regulatory states without DNA breakage	Persistence, reversibility, and cell‐state dependence remain uncertain	Ectopic epigenetic alteration, long‐term regulatory imbalance, and lineage‐specific effects

*Note*: Superscript numbers indicate representative references for the corresponding category.

### 5.5 Significance of double‐strand break‐free editing strategies for genome stability and safety

The treatment of genetic diseases typically pursues long‐term benefit and high safety; therefore, reducing risks associated with double‐strand breaks has become an important direction in the development of precision editing technologies.^7^ Studies indicate that^43^, ^44^ DNA double‐strand breaks may induce large deletions, chromosomal translocations, complex rearrangements, and DNA damage responses, which are particularly concerning in therapeutic editing scenarios. Existing studies suggest that strategies such as base editing, prime editing,^16^, ^23^, ^25^, ^58^ CRISPRi, CRISPRa, and epigenetic editing can reduce or avoid classical double‐strand breaks to varying degrees, thereby minimizing perturbations to genome stability. For non‐regenerative tissues, terminally differentiated cells, or diseases requiring long‐term in vivo safety, double‐strand break‐free strategies hold high theoretical appeal.

**FIGURE 4:**
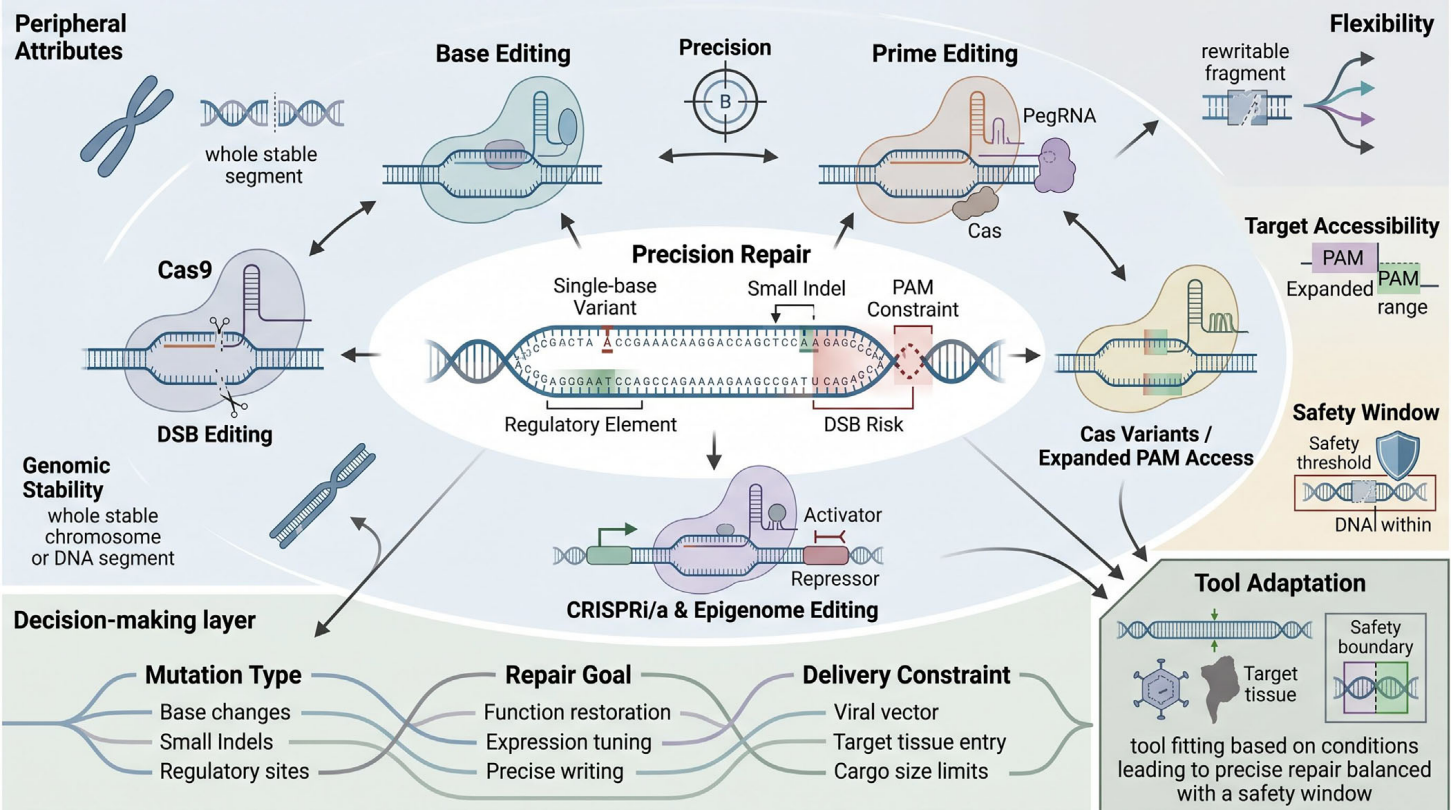
Complementary and adaptive relationships among CRISPR‐derived precision editing tools.

Double‐strand break‐free does not imply complete absence of risk. Available evidence indicates that base editing can produce bystander edits or deaminase‐related unintended changes; prime editing^45^, ^48^, ^58^, ^59^, ^60^ may be accompanied by byproducts or uneven efficiency, and CRISPR transcriptional regulation and epigenetic editing require attention to regulatory persistence, reversibility, and cell‐state dependence. Therefore, the evaluation of precision editing strategies should extend from ‘whether double‐strand breaks occur’ to a comprehensive assessment of editing accuracy, functional restoration, genome stability, and long‐term safety. Thus, different precision editing tools do not exhibit a linear replacement relationship; instead, they form complementary relationships based on mutation type, repair goal, delivery conditions, and safety boundaries. The key to repairing pathogenic mutations in genetic diseases lies not in selecting a single ‘optimal tool’, but in establishing an editing strategy matched to the specific variant mechanism (Figure 4).

## 6 IMPLEMENTATION STRATEGIES FOR REPAIRING PATHOGENIC MUTATIONS IN GENETIC DISEASES: EX VIVO EDITING, IN VIVO DELIVERY, AND DISEASE‐TYPE ADAPTATION

### 6.1 Ex vivo gene editing strategies: Haematopoietic stem cells, iPSCs, and differentiated cells

The translational advantage of ex vivo editing stems from the controlled environment, which allows for cell expansion, edited product characterization, clonal or population screening, and pre‐infusion quality control. Haematopoietic stem cells (HSCs) represent one of the most mature cell types for ex vivo editing, as they can be harvested, processed, and reinfused, while possessing long‐term haematopoietic reconstitution potential. For hemoglobinopathies, certain immunodeficiencies, and haematological metabolic disorders, available evidence generally supports^7^, ^61^, ^62^ ex vivo editing as an important approach to mitigate the risks of uncontrolled in vivo editing. However, this controllability is accompanied by complex cell manufacturing processes, batch consistency control, pre‐infusion conditioning, and patient preparative burdens; thus, the translational benefit must be evaluated alongside production costs, treatment logistics, and clinical accessibility.

The ex vivo approach is not limited to the haematopoietic system; its application boundaries are expanding with the development of iPSC and cell differentiation technologies.^35^, ^42^ iPSCs can be corrected for pathogenic mutations and then differentiated into disease‐relevant cell types, offering potential cell replacement strategies for inherited neurological diseases, cardiomyopathies, liver diseases, and retinal disorders. Nevertheless, iPSC‐derived cells still face translational challenges, including differentiation purity, maturity, residual pluripotent cells, tumorigenic risk, and immune matching. For terminally differentiated cells, ex vivo editing facilitates quality control, but limited expansion capacity, post‐implantation survival, and tissue integration efficiency may become major bottlenecks. Taken together, ex vivo editing is most suitable when disease‐relevant cells can be safely collected, expanded or maintained, quality‐tested, and reinfused with measurable endpoints; however, this framework is less applicable when target cells cannot be harvested, have poor engraftment potential, or depend on an intact in vivo niche.^35^ Therefore, its advantage lies not only in controllable editing but also in feasible pre‐infusion quality assessment; its limitations extend beyond technical complexity to the combined translational barriers of cell manufacturing, conditioning regimens, and treatment centre capabilities.

### 6.2 In vivo editing strategies: target tissue selection and delivery barriers

For target cells that are difficult to harvest, expand, or reinfuse, in vivo editing offers the possibility of directly completing pathogenic mutation repair in diseased tissues.^7^, ^63^ The liver, eye, muscle, and central nervous system are among the most investigated tissues in in vivo editing research. The liver benefits from abundant blood flow and relative accessibility to nanoparticle delivery, while the eye offers advantages owing to local administration and immune privilege. Existing studies suggest that certain metabolic genetic diseases, inherited retinal disorders, and neuromuscular diseases could be treated by the in vivo delivery of editing systems to correct pathogenic sequences or aberrant expression states in affected tissues.

The major limitations of in vivo editing arise from delivery barriers and tissue‐specific control.^28^ Studies indicate that^59^, ^60^ tissues differ in vascular permeability, cellular uptake capacity, immune microenvironment, and cell turnover rates, all of which influence the probability that the editing system reaches target cells and mediates a productive modification. The blood–brain barrier, the large volume of muscle tissue, the mucus barrier in the lung, and renal filtration characteristics can all reduce delivery efficiency or increase dose requirements.^64^ Related studies also suggest that therapeutic in vivo delivery of gene‐editing agents is generally less amenable to correction through cell selection compared with ex vivo editing.^65^ Therefore, in vivo strategies must simultaneously consider tissue targeting, expression duration, dose safety windows, and long‐term monitoring.

### 6.3 Application of AAV, lipid nanoparticle, and non‐viral delivery systems in treating genetic diseases

Delivery systems largely determine whether CRISPR‐mediated repair can be translated from molecular design into functional tissue benefits.^66^ Due to their relatively defined tissue tropism, extensive in vivo transduction experience, and a substantial body of long‐term research, AAV vectors are widely used in gene therapy studies targeting the eye, liver, muscle, and nervous system. For CRISPR systems, AAV can deliver a Cas coding sequence, an sgRNA, or a donor template, but its limited packaging capacity may restrict the accommodation of larger Cas variants, prime editing^66^ components, or complex regulatory elements. Related studies further indicate that AAV‐associated immune responses, pre‐existing neutralizing antibodies, and the risk of sustained editing from long‐term expression are critical factors that must be evaluated when designing treatments for genetic diseases.

**FIGURE 5:**
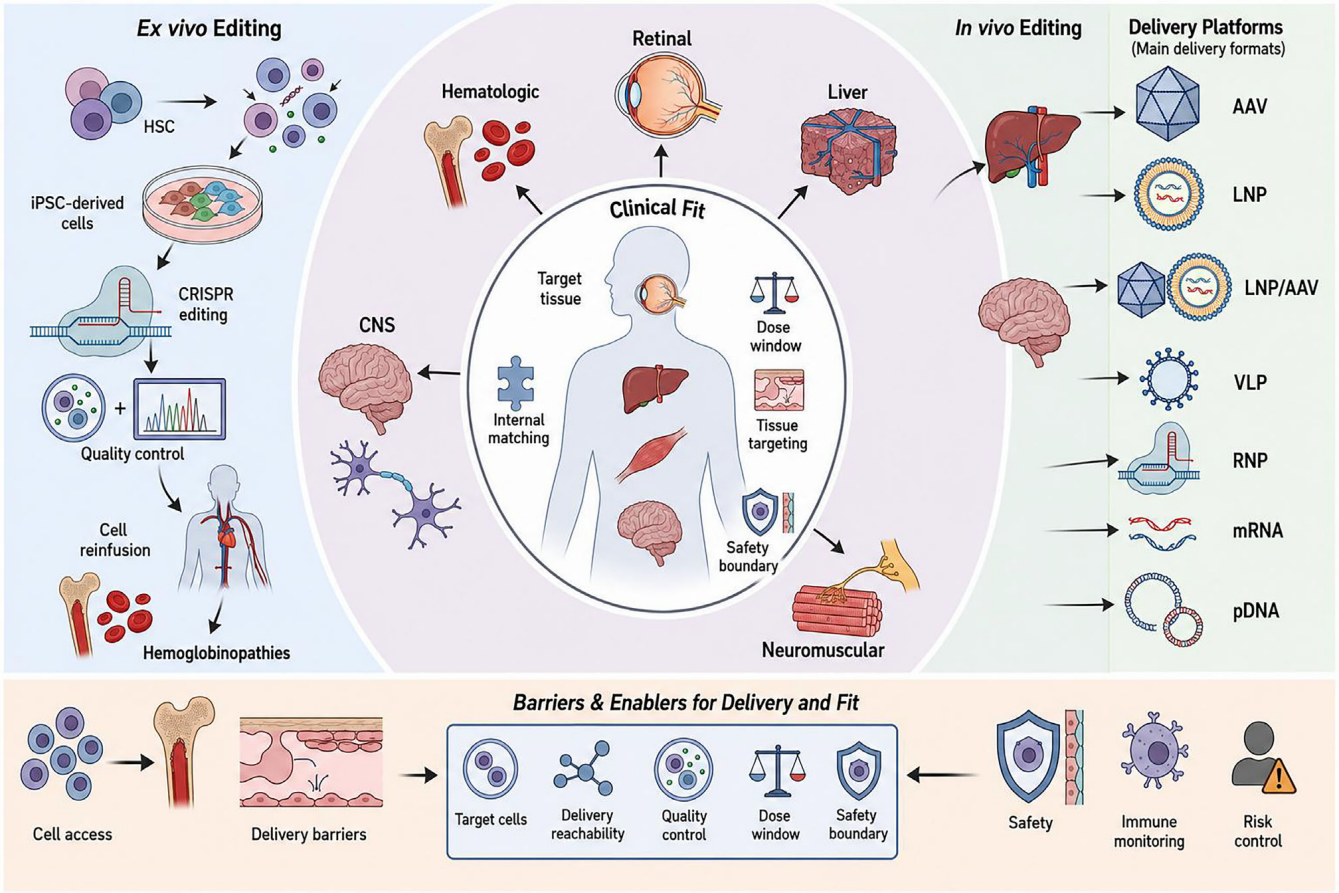
Ex vivo editing, in vivo delivery, and strategy alignment with genetic disease types.

LNP and other non‐viral systems provide an important complement for transient expression‐type editing. Studies have reported that^67^, ^68^ LNP can deliver mRNA, sgRNA, or ribonucleoprotein complexes, with characteristics of relatively short expression duration, scalable production, and flexible design. Studies in liver‐targeted editing suggest that LNP is particularly suitable for scenarios requiring short‐term exposure to editing tools. In addition to LNP, polymer nanoparticles, virus‐like particles, extracellular vesicles, and physical delivery technologies have also been discussed as potential approaches. Recent delivery research further tends to improve cell selectivity through tissue‐targeting lipid components, virus‐like particles, engineered extracellular vesicles, transient ribonucleoprotein delivery, and peptide‐ or antibody‐conjugated modification systems,^67^, ^69^, ^70^ to reduce the risk of systemic exposure and off‐target effects in non‐target tissues. Overall, the choice of delivery system essentially involves trade‐offs among tissue tropism, expression duration, cargo capacity, immunogenicity risk, and the ability for repeated administration (Figure 5).^68^, ^71^

Delivery choice should also be matched to the physical format of the editing cargo. Plasmid DNA can provide sustained expression but raises concerns about prolonged nuclease exposure and integration risk; mRNA plus guide RNA enables transient expression and is compatible with LNP manufacturing, but requires careful optimization of RNA chemistry, dose, and innate immune activation; RNP delivery offers the shortest exposure window and is commonly used in ex vivo electroporation, although cellular toxicity and recovery rates may limit some products. Large editors create additional constraints: prime editors may require dual‐AAV or split systems for in vivo delivery, whereas compact Cas variants, transient LNP‐mRNA, or VLP/RNP formats may be preferable when single‐cargo capacity, exposure duration, or GMP scalability is decisive. Therefore, delivery architecture should be assessed together with cargo size, expression duration, target tissue, product‐release testing, and manufacturing feasibility.

A practical delivery matrix should therefore evaluate the editing vehicle and the editing architecture together. AAV is attractive for local or tissue‐tropic delivery but is constrained by packaging capacity, making large prime editors or multi‐component systems difficult unless dual‐AAV or split‐intein designs are used. LNP‐mRNA platforms allow transient nuclease or editor expression and scalable manufacturing, but current extrahepatic targeting remains less mature than liver delivery. RNP and VLP formats shorten exposure time and may reduce persistent nuclease activity, whereas pDNA can support longer expression but raises concerns about prolonged editing windows and potential integration. Compact Cas variants may improve single‐cargo AAV compatibility, while prime editors require particular scrutiny because their size and pegRNA requirements create major in vivo delivery barriers.

### 6.4 Factors influencing editing efficiency, tissue specificity, persistence, and dose control

The therapeutic significance of mutation repair in genetic diseases cannot be judged solely by editing efficiency; rather, it depends on whether the edited cells are sufficient to restore critical tissue function. Studies have reported that^7^, ^71^ different diseases have varying requirements for the editing threshold: in some secretory protein deficiency diseases, restoring function in a low proportion of cells may bring systemic benefits, whereas structural protein defects, neuromuscular diseases, or developmental abnormalities often require more extensive tissue‐level repair. Evidence suggests that editing efficiency is also jointly influenced by target cell renewal capacity, mutation type, chromatin accessibility, and delivery uniformity.

Tissue specificity, expression persistence, and dose control collectively determine the safety margin of an editing strategy.^28^ Studies have reported that^59^, ^60^, ^68^, ^72^ insufficient dose may fail to achieve functional recovery, while excessive dose may increase the risk of immune responses, non‐target tissue exposure, and editing byproducts. For in vivo editing, sustained expression of editing tools may increase the chance of modifying the target site, but it may also prolong the window of off‐target effects and immune exposure; transient expression helps reduce long‐term risks, but it places higher demands on delivery efficiency. Overall, the ideal strategy should achieve a balance among editing efficiency, tissue selectivity, expression duration, and the dose‐safety window, rather than simply pursuing the highest editing percentage.

### 6.5 Comparative analysis of ex vivo and in vivo repair strategies in different types of genetic diseases

Different genetic diseases show varying suitability for ex vivo and in vivo editing.^7^ Studies have reported that haematological genetic diseases are generally more suitable for ex vivo editing, because haematopoietic stem cells can be collected, tested, and reinfused, and they can support long‐term efficacy through haematopoietic reconstitution. Evidence suggests that^26^ the advantage of this approach is sufficient quality control, but it also involves conditioning toxicity, cell manufacturing costs, and accessibility issues. Ophthalmic genetic diseases are more suitable for local in vivo delivery, because the ocular structure is relatively enclosed, the administered dose is lower, and efficacy evaluation is more direct; liver genetic diseases also have potential for in vivo editing, but attention must be paid to the delivery system, immune responses, and long‐term expression control.

The strategic choices for neuromuscular diseases and central nervous system genetic disorders are more complex. Studies suggest that these diseases often involve widespread tissue distribution, non‐regenerative cells, or special barrier structures, thereby making in vivo delivery more challenging, while ex vivo cell replacement struggles to fully restore tissue network function. Muscle diseases may require extensive tissue delivery, while nervous system diseases must address the blood‐brain barrier, cell‐type selection, and long‐term safety. Overall, ex vivo and in vivo editing should not be viewed as simply opposing pathways but should be matched based on target tissue accessibility, functional recovery thresholds, delivery risks, and clinical implementation conditions. Future mutation repair for genetic diseases will more likely rely on disease‐type‐customized integrated editing‐delivery‐evaluation strategies. Therefore, the core advantage of ex vivo editing lies in quality control and cell selection, while that of in vivo editing lies in directly acting on diseased tissues; the choice between the two depends essentially on target cell availability, functional recovery thresholds, delivery risks, and clinical implementation complexity.

## 7 MULTI‐DIMENSIONAL EVALUATION OF FUNCTIONAL RESTORATION AND PHENOTYPIC REMODELLING AFTER GENE EDITING

### 7.1 Molecular level: mutation correction, gene expression restoration, and protein function reconstruction

Evaluation of gene editing for genetic diseases cannot remain at the single level of whether the target sequence has been modified but should further determine whether molecular function is restored after correction of pathogenic mutations.^4^ Studies indicate that CRISPR‐related editing technologies can confirm target‐site changes by sequencing, yet sequence‐level correction does not necessarily equate to recovery of RNA expression, protein products, and pathway activity. For loss‐of‐function mutations, related research typically focuses on whether the coding sequence is restored, whether mRNA is stable, whether protein expression reappears, and whether key domains are intact. For gain‐of‐function or dominant‐negative mutations, studies suggest that evaluation should shift to whether abnormal transcripts, aberrant protein activity, or pathological molecular interactions are attenuated.

Molecular functional recovery also requires distinguishing between correct editing outcomes and effective biological output. Previous research suggests that, even when target bases or mutation sites are accurately rewritten, splicing patterns, RNA degradation, translation efficiency, protein folding, subcellular localization, and post‐translational modifications can still affect the ultimate functional output. For non‐coding region mutations, evaluation should extend to transcription factor binding, enhancer activity, chromatin accessibility, and target gene expression changes. Molecular‐level evaluation should establish a hierarchical chain of evidence from DNA sequence, RNA expression, protein products to pathway activity, and position editing efficiency as the starting point, not the endpoint, of functional recovery assessment.

### 7.2 Cellular level: cell fate, signalling pathways, and disease‐associated phenotypic improvement

Functional abnormalities in genetic diseases ultimately often manifest as alterations in cell state, cell fate, and specific physiological functions; therefore, post‐editing evaluation needs to extend from molecular correction to phenotypic remodelling at the cellular level.^35^, ^42^ Studies indicate that iPSCs and their differentiation models, primary cell models, and organoid systems can be used to analyze the impact of genetic background on cell differentiation, maturation, and disease‐related phenotypes. For genetic diseases of the nervous system, myocardium, liver, retina, or haematopoietic system, related research commonly considers differentiation capacity, metabolic state, stress response, electrophysiological properties, secretory function, or cell survival as important references for functional recovery. This evaluation approach helps determine whether gene editing truly alters disease‐related cell states, rather than merely achieving local sequence rewriting.

Cellular phenotypic rescue exhibits marked context dependence, which is particularly prominent in complex genetic diseases and developmental disorders. Evidence suggests that certain pathogenic mutations may cause only subtle abnormalities under standard culture conditions, but display clearer pathological features at specific differentiation stages, in stress environments, or in three‐dimensional organoid systems. For such cases, relevant studies emphasize the need to combine disease‐relevant stimuli, developmental time windows, and cell‐type‐specific readouts to evaluate post‐editing changes. Therefore, cellular‐level evaluation should focus more on whether disease‐related readouts, such as cell fate, stress response, metabolic function, electrophysiological activity, or secretory capacity, return to a normal state after mutation correction.

### 7.3 Single‐cell transcriptomics dissects cell state changes after editing

As genetic disease research shifts from average expression changes to cellular heterogeneity and state transitions, single‐cell transcriptomics has become an important tool for evaluating functional recovery after editing. Studies indicate that^9^, ^73^ by combining CRISPR perturbation with single‐cell transcriptome readouts, it is possible to link gene perturbation information with cell‐state changes within the same system, thereby dissecting how specific genes or mutations affect transcriptional networks. For disease models after gene editing, relevant studies suggest that such methods can help determine whether mutation correction reduces abnormal cell subpopulations, restores differentiation trajectories, or re‐establishes key regulatory modules. Compared with bulk transcriptomics, single‐cell analysis is more suitable for identifying rare cell populations, transitional states, and incomplete recovery states.

Single‐cell evaluation also reveals that functional recovery after editing does not always occur uniformly. Single‐cell studies show^9^, ^74^ that cellular responses after CRISPR perturbation may exhibit significant heterogeneity, and average expression changes may mask important subpopulation differences. Such differences may arise, on one hand, from technical factors such as editing products, perturbation efficiency, sample processing, and sequencing readouts, and, on the other hand, may reflect true biological heterogeneity caused by cell state, developmental stage, microenvironment, and lineage bias. Methodological studies further indicate that perturbation omics systems such as Perturb‐seq need to distinguish true biological responses from technical factors, such as incomplete editing, cell‐cycle differences, clonal expansion, or sample processing biases. Therefore, the value of single‐cell transcriptomics lies not only in evaluating gene function recovery but also in identifying residual abnormal cell states and potential risk signals that are difficult to capture by population average analysis.

### 7.4 Epigenomics, spatial omics, and proteomics reveal regulatory network reconstruction

Gene function imbalance in genetic diseases often involves chromatin state, spatial organizational structure, and protein interaction networks, and a single transcriptional readout is insufficient to fully explain functional recovery after editing. Studies indicate that^75^ CRISPR perturbation studies have expanded from the transcriptome to chromatin accessibility, epigenetic modifications, spatial transcriptomics, and multi‐modal single‐cell omics to dissect the impact of gene alterations at different regulatory levels. For noncoding mutations, enhancer abnormalities, or developmental regulatory disorders, relevant studies suggest that epigenomics helps determine whether editing restores the open chromatin state and regulatory element activity. Spatial omics can place expression changes back into the tissue structural context and can be used to assess whether cell location, neighbour interactions, and the local microenvironment are synchronously improved.

Proteomics and functional protein readouts can further compensate for the shortcomings of transcriptional‐level evaluation. Evidence suggests that^75^, ^76^, ^77^ mRNA recovery does not necessarily represent the complete restoration of protein abundance, post‐translational modifications, complex assembly, or signalling pathway function, especially in metabolic diseases, structural protein disorders, and diseases with aberrant signal transduction, where protein‐level evidence requires greater attention. For CRISPR‐edited models, relevant studies, through proteomics, phosphoproteomics, and interactomics analyses, can identify pathways that tend to normalize after mutation correction and can also reveal downstream networks that remain dysregulated. Taken together, multi‐omics evaluation is driving the judgment of functional recovery from ‘whether a single gene is repaired’ to ‘whether the regulatory network is reconstructed’.

### 7.5 Multi‐omics integration evaluates gene function recovery and long‐term tissue homeostasis

Following evaluation at molecular, cellular, and tissue levels, the key role of multi‐omics integration lies in assessing the consistency among readouts from different layers, rather than reconfirming the editing event itself. Studies indicate that combining CRISPR with sequencing, single‐cell omics, epigenomics, and proteomics can integrate genetic variants, editing outcomes, and functional readouts into a continuous evidence chain. For pathogenic mutation correction, multi‐omics integration enables simultaneous assessment of DNA‐editing results, RNA expression changes, chromatin status, protein function, cellular composition, and tissue architecture. This framework expands the evaluation of editing efficacy beyond single endpoints, providing a systematic judgment closer to the authentic pathological process of genetic diseases.

**FIGURE 6:**
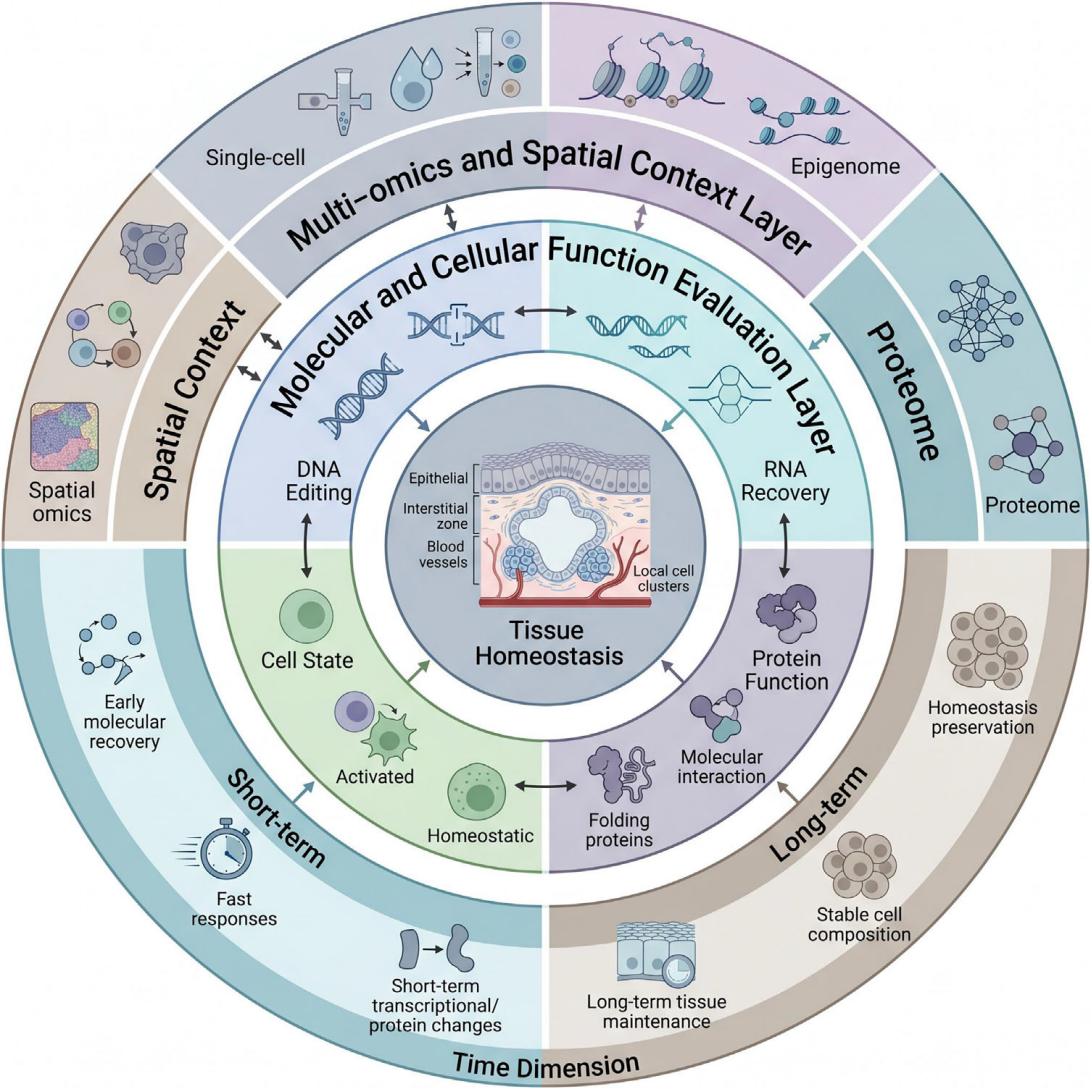
Multi‐dimensional evaluation system for functional restoration and phenotype remodelling after gene editing.

Long‐term tissue homeostasis assessment represents the most overlooked yet critical dimension of functional recovery. Evidence suggests that^35^, ^39^, ^60^, ^78^ iPSC‐derived models, organoid systems, and in vivo models can be used to analyze cell differentiation, tissue architecture, and long‐term functional changes, although their maturity, batch variation, and model integrity may still constrain the boundaries of interpretation. For in vivo editing or clinical translation scenarios, relevant studies emphasize the need to consider tissue renewal, immune responses, cell competition, clonal expansion, and age‐related changes. Therefore, the ultimate goal of a multi‐dimensional evaluation system is not to prove that a specific editing event has occurred, but to determine whether the molecular network, cellular state, and tissue homeostasis exhibit a directionally consistent and long‐term stable recovery trend following mutation correction (Figure 6).

## 8 SAFETY, OFF‐TARGET EFFECTS, AND IMMUNOGENICITY ISSUES IN CRISPR GENE EDITING

### 8.1 Molecular sources, detection methods, and evaluation criteria of off‐target effects

When CRISPR gene editing enters the therapeutic context of genetic diseases, targeting accuracy becomes the primary safety concern determining its translational potential.^26^ Studies indicate that off‐target effects mainly arise from partial complementarity between sgRNA and non‐target genomic sequences, potentially causing Cas proteins to generate unintended cleavage or base modification at similar sequences. The unintended risks associated with different editing tools are not identical: nuclease‐based Cas9 systems primarily focus on cleavage‐type off‐target events, insertions/deletions, and structural variants; base editing requires greater assessment of bystander editing and deaminase‐associated DNA or RNA alterations; prime editing^26^, ^79^, ^80^ demands attention to unintended byproducts, editing product purity, and site‐dependent differences. This risk is jointly influenced by sgRNA design, the degree of PAM‐proximal sequence matching, chromatin accessibility, Cas protein activity, editing tool exposure time, and cell type. For the treatment of genetic diseases, if off‐target sites fall within tumour suppressor genes, proto‐oncogenes, splicing regulatory regions, or critical enhancer regions, their potential consequences should be considered as high‐priority risks.

Regarding off‐target evaluation, technical systems have expanded from candidate site prediction to comprehensive genome‐wide detection.^79^, ^80^, ^81^ Methodological studies indicate that in vitro cleavage assays, intracellular capture assays, targeted deep sequencing, whole‐genome sequencing, and long‐read sequencing can identify non‐target editing from different perspectives. Research suggests that a single method cannot cover all types of off‐target events; therefore, high‐risk editing strategies typically require a combination of computational prediction, unbiased screening, and confirmatory sequencing. Taken together, current research suggests that off‐target evaluation should not be limited to whether non‐target editing occurs, but must also assess its frequency, genomic location, functional consequences, and the safety risk after the expansion of edited cells.

### 8.2 Methodological assessment of off‐target effects, product heterogeneity, and genotoxicity

Current risk assessment should combine complementary assays rather than rely on a single detection technology. GUIDE‐seq can capture double‐strand break‐associated off‐target events in living cells, whereas CHANGE‐seq, CIRCLE‐seq, and Digenome‐seq provide highly sensitive in vitro or cell‐free screening of nuclease activity. Targeted amplicon sequencing and whole‐genome sequencing can quantify candidate‐site editing, while long‐read sequencing is particularly useful for large deletions, complex alleles, and structural variants. Single‐cell sequencing helps resolve cell‐to‐cell heterogeneity, edited subclones, and lineage‐specific responses after editing; karyotyping, copy‐number analysis, ddPCR, and orthogonal structural variant assays remain important for detecting chromosomal abnormalities. Each method has limitations, including differences between in vitro and intracellular activity, detection thresholds for low‐frequency events, incomplete coverage of rare structural outcomes, and limited ability to predict long‐term clonal expansion.

### 8.3 Risks of editing product heterogeneity, DNA double‐strand breaks, and chromosomal structural abnormalities

CRISPR‐edited cell populations typically do not consist solely of a single intended repair product; instead, they comprise a mixture of unedited cells, correctly repaired cells, unintended indel byproducts, bystander‐edited cells, cells with large deletions, cells with chromosomal structural abnormalities, and cells with distinct editing outcomes across multiple alleles.^34^ For mutation repair in genetic diseases, this heterogeneity of editing products directly affects efficacy assessment and safety evaluation. Specifically, the proportion of intended repair determines the likelihood of functional recovery, while unintended editing products, aberrant allelic combinations, or structural byproducts may introduce new functional perturbations. Therefore, editing efficiency should not be represented solely by the overall editing rate; it is necessary to further distinguish among different product types, including intended repair, unedited products, indel byproducts, bystander editing, and structural variants.

Building upon the aforementioned product heterogeneity, canonical CRISPR‐Cas9 relies on DNA double‐strand breaks to achieve gene knockout or precise repair; however, double‐strand breaks themselves may also become a significant source of genomic instability. Studies indicate that^33^, ^43^, ^82^ Cas9 cleavage can lead to unintended insertions/deletions, large deletions, inversions, translocations, and complex chromosomal rearrangements. For haematopoietic stem cells, progenitor cells, or other cells with long‐term proliferative potential, such structural alterations may persist after editing and be maintained during cell expansion, clonal selection, or post‐infusion, thereby affecting subsequent safety. Related studies also suggest that different genomic regions and cell types exhibit distinct DNA repair propensities; therefore, the product spectrum of the same editing tool may differ across disease models.

DNA damage responses further complicate safety evaluation. Previous studies observed that Cas9‐induced double‐strand breaks can activate p53‐related pathways, cell cycle checkpoints, and apoptosis or senescence programs. This trend can be interpreted as a protective cellular response to genomic damage; however, under certain editing conditions, such stress may also alter the composition of the cell population, conferring a relative selective advantage to cells with specific damage response characteristics and further amplifying the heterogeneity of the edited cell population. Methodological guidance emphasizes^83^, ^84^, ^85^ that ex vivo editing cell products should incorporate on‐target product spectrum analysis, bystander editing detection, large‐deletion screening, karyotyping, copy‐number variation detection, structural‐variant assessment, clonal composition analysis, and evaluation of long‐term expansion stability. Overall, the safety of double‐strand break editing cannot be judged solely by whether the intended target site is correctly rewritten; it is also necessary to examine editing product composition, genomic structural integrity, and cellular stress status.

In addition, clinically oriented genotoxicity evaluation should explicitly distinguish small indels from larger on‐target and genome‐wide structural outcomes. Chromothripsis‐like fragmentation, megabase‐scale truncations, inversions, translocations, and on‐target large deletions may be missed if assessment is limited to short‐read amplicon sequencing around the cut site. This concern is especially relevant for haematopoietic stem cells, iPSCs, and other proliferative cell products because rare structural events can become enriched during culture, selection, or post‐treatment expansion. Accordingly, DSB genotoxicity assessment should integrate product‐spectrum analysis, structural variant detection, long‐read sequencing, cytogenetic evaluation, and clonal tracking before therapeutic conclusions are drawn.

### 8.4 Immune responses associated with Cas proteins, sgRNA, and delivery vectors

When gene editing systems enter the body, their safety depends not only on the accuracy of genomic rewriting but also on host immune recognition. Some Cas proteins derive from common bacterial orthologs, and prior microbial exposure may create pre‐existing humoral and cellular immunity, including neutralizing antibodies and CD4+ or CD8+ memory T‐cell responses against Cas9‐related proteins. For in vivo editing, Cas protein expression duration, dosage level, tissue distribution, and delivery modality may influence whether immune recognition causes clearance of edited cells, local inflammation, reduced therapeutic efficacy, or limitations on re‐dosing. Potential mitigation strategies include minimizing nuclease exposure time, using transient RNA/RNP delivery, selecting less immunogenic orthologs, engineering Cas variants, and exploring computational de‐immunization approaches, although these strategies require careful validation before clinical generalization.

This issue is particularly important because several Cas nucleases originate from bacterial species to which humans may have prior exposure. Pre‐existing neutralizing antibodies may reduce exposure of the editing system to target cells, whereas CD4+ and CD8+ memory T‐cell responses may eliminate cells that transiently or persistently express bacterial Cas proteins. AAV delivery adds a second immunological layer through capsid‐directed antibodies, complement activation, and T‐cell responses, and repeated dosing may be difficult when vector immunity is strong. Engineering approaches such as transient mRNA or RNP delivery, reduced‐expression windows, ortholog selection, protein engineering, and computational de‐immunization may reduce immunogenicity, but they must be evaluated alongside editing efficiency and tissue‐specific safety.

Delivery vectors and nucleic acid components may also contribute to immunogenic risk. Studies indicate that AAV vectors can be affected by neutralizing antibodies, complement activation, and T cell responses, whereas LNPs may induce innate immune responses, inflammatory cytokine release, or hepatic stress. sgRNA, mRNA, and other nucleic acid components may also trigger immune signalling through pattern recognition receptors. Overall,^86^ immunogenicity assessment needs to simultaneously cover Cas proteins, nucleic acid components, delivery vectors, and the target tissue microenvironment. For genetic diseases requiring repeated administration or high‐dose delivery, immune memory, feasibility of re‐administration, and chronic inflammation risk need to be particularly incorporated into strategy design.

### 8.5 Long‐term safety, potential oncogenic risks, and boundaries of germline editing

Gene editing for genetic diseases usually aims for long‐term or even lifelong benefits; therefore, short‐term safety results are insufficient to support a complete risk evaluation. Studies indicate that^87^ edited cells may persist for a long time in the body, especially haematopoietic stem cells, hepatocytes, muscle cells, and nervous system cells, which have durable functional significance. If low‐frequency harmful mutations, chromosomal abnormalities, or clonal expansion dominance arise during editing, their impact may not become apparent until much later. Therefore, related studies emphasize that long‐term follow‐up should focus on the durability of efficacy, clonal dynamics, tumour‐related events, immune responses, and tissue function changes.

Potential oncogenic risk is one of the most concerning long‐term issues in therapeutic editing. Evidence suggests that^32^ if off‐target editing affects tumour suppressor genes, DNA repair genes, cell cycle regulators, or regions of chromosomal stability, it may theoretically increase the risk of abnormal clonal expansion. Regarding germline editing, international ethical and regulatory guidelines generally emphasize that its risks involve not only the edited individual but also their offspring and the population's genetic structure; hence, it should not be conflated with somatic therapeutic editing. Under current ethical and regulatory frameworks, discussions on the clinical translation of therapeutic gene editing for genetic diseases must be explicitly limited to somatic editing, clearly distinguishing it from the ethical controversies and intergenerational risks associated with germline editing.^88^

### 8.6 Engineering strategies and risk control framework to enhance editing safety

Faced with multi‐level safety challenges, CRISPR technology development is shifting from an exclusive pursuit of editing efficiency towards a balance of efficiency, precision, and controllability. Available evidence indicates that strategies such as utilizing high‐fidelity Cas9 variants, optimizing sgRNA design, reducing exposure time to editing tools, delivering ribonucleoprotein (RNP) complexes, and employing tissue‐specific promoters can help mitigate off‐target effects and long‐term expression risks.^89^, ^90^ For point mutation repair, studies regard strategies that avoid classic double‐strand breaks, such as base editing and prime editing,^50^, ^90^ as critical directions for minimizing genomic structural damage.

Safety control should be integrated into the entire process of editing design, delivery implementation, product detection, and long‐term follow‐up. Methodological guidance emphasizes^90^ that developing therapeutic CRISPR applications requires establishing a stratified risk assessment system, including in vitro off‐target screening, genome‐wide structural variant detection, cell function evaluation, immunogenicity analysis, animal model validation, and long‐term clinical monitoring. Therefore, safety must not be treated as an afterthought before clinical translation, but rather as an integral component of the editing strategy design. For genetic diseases, an optimal risk‐control framework must simultaneously determine whether the editing is accurate, whether the cells are stable, whether the immune response is controllable, and whether the long‐term benefits outweigh the potential risks.

**FIGURE 7:**
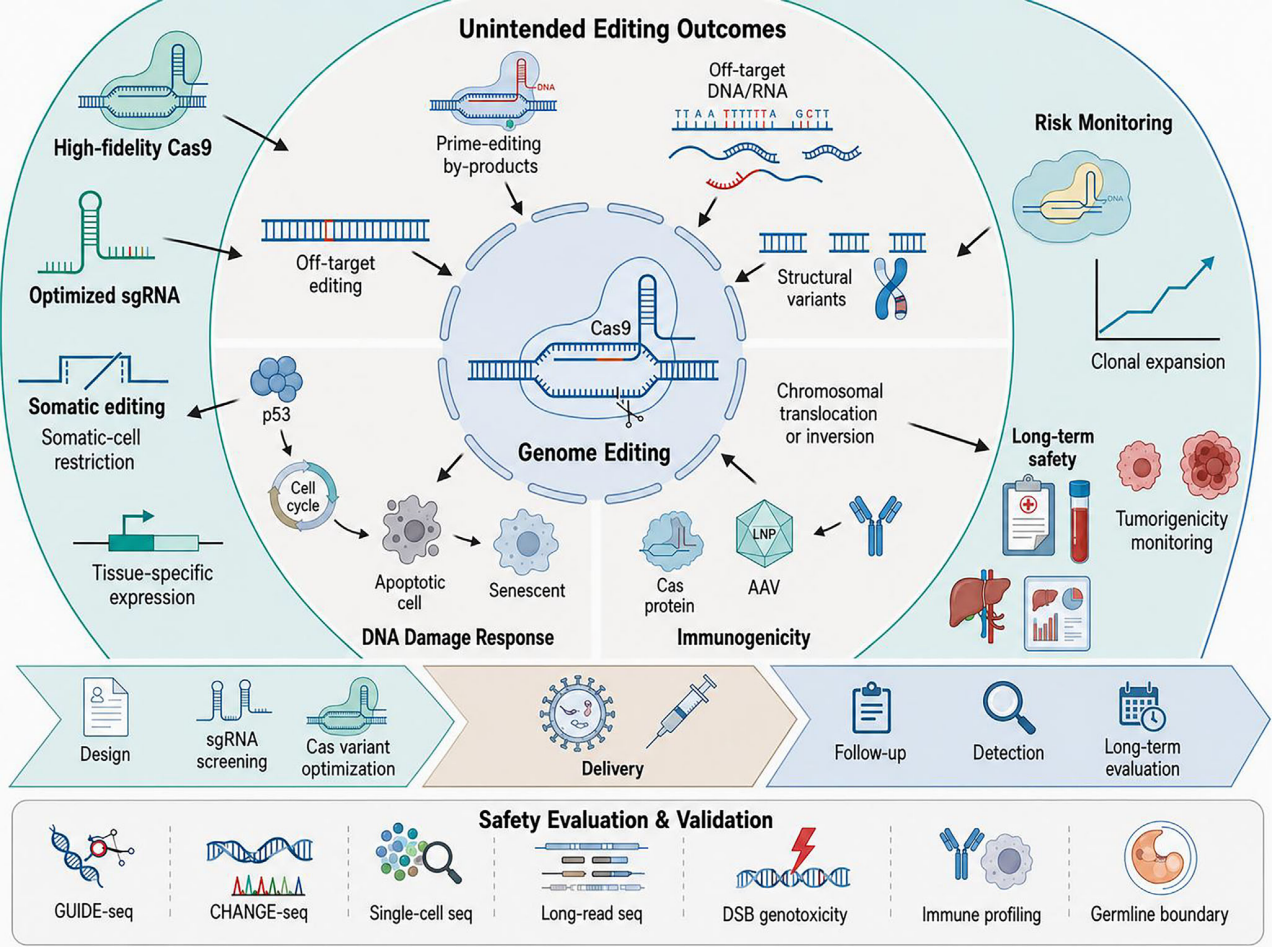
Major sources of safety risks in CRISPR gene editing and the corresponding simplified risk‐control framework.

These strategies collectively form a multi‐layered risk‐control framework for CRISPR‐based therapeutic gene editing, integrating editing accuracy, genomic stability, delivery optimization, immunogenicity assessment, somatic‐cell restriction, and long‐term safety monitoring (Figure 7).

## 9 CLINICAL TRANSLATION PROGRESS, REAL‐WORLD CHALLENGES, AND FUTURE DIRECTIONS OF CRISPR‐Cas9 IN GENETIC DISEASES

### 9.1 Progress of CRISPR gene therapy for genetic diseases from clinical trials to approved applications

Casgevy (exagamglogene autotemcel; exa‐cel)^91^, ^92^ CRISPR‐based gene therapy for genetic diseases has achieved the transition from proof of concept to regulatory approval in selected haematologic indications. represents the clearest example. The FDA first approved Casgevy on 8 December, 2023, for patients aged 12 years and older with sickle cell disease and recurrent vaso‐occlusive crises, and subsequent FDA labelling includes sickle cell disease and transfusion‐dependent β‐thalassemia; a supplemental FDA approval in July 2026 expanded eligibility to patients aged 2 years and older with sickle cell disease or transfusion‐dependent β‐thalassemia. In the European Union, Casgevy received conditional marketing authorization on 9 February, 2024, for transfusion‐dependent β‐thalassemia and severe sickle cell disease in patients aged 12 years and older when haematopoietic stem‐cell transplantation is appropriate and no suitable matched related donor is available. These regulatory milestones show that clinical translation is no longer theoretical, but they also define a narrow evidence base centred on ex vivo haematopoietic editing rather than broad readiness across all genetic diseases.

**FIGURE 8:**
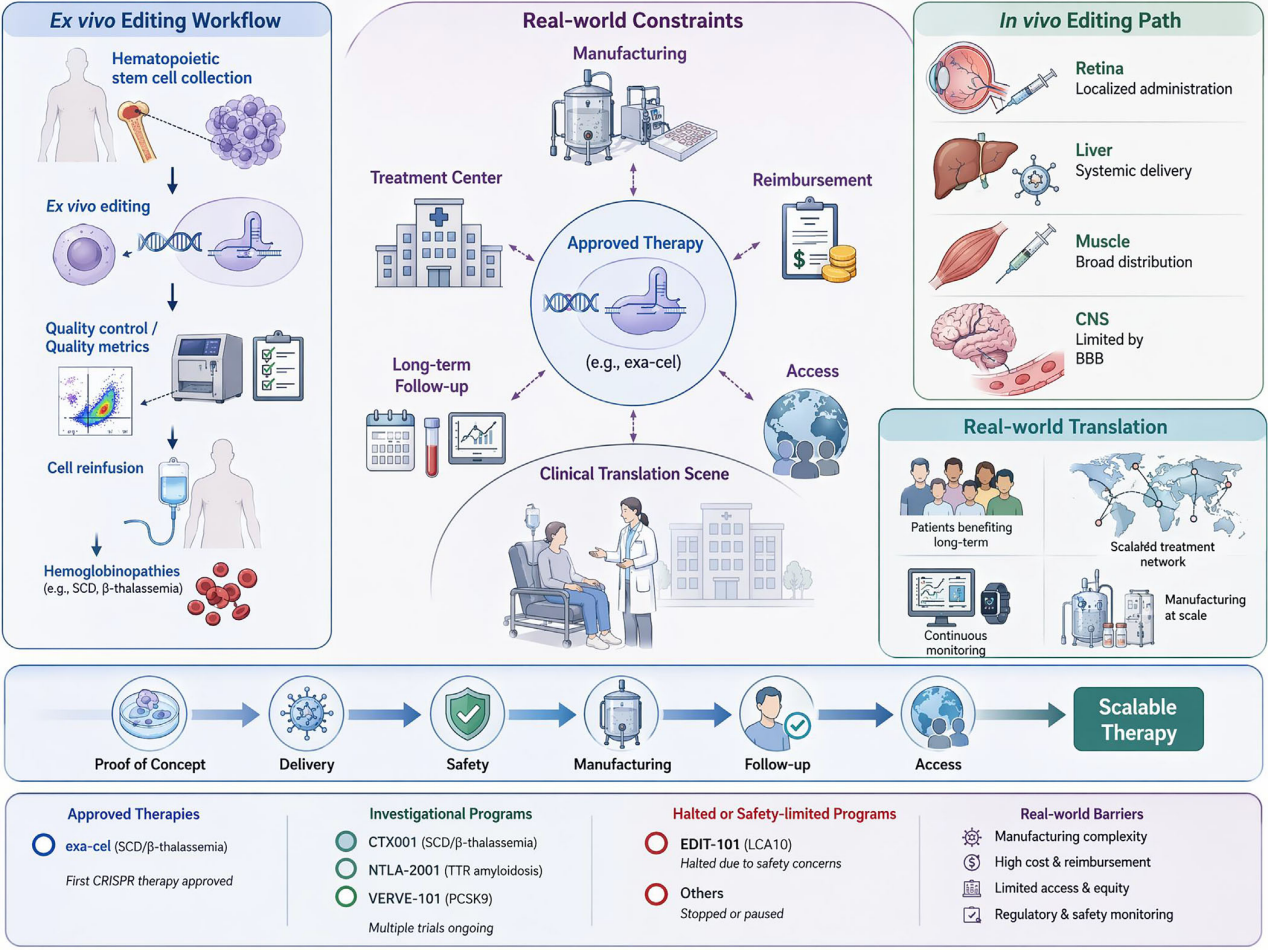
Clinical translational landscape and practical barriers of CRISPR therapies for genetic diseases.

The clinical landscape should therefore be interpreted through an evidence hierarchy rather than as a single continuum. Approved ex vivo haematopoietic editing products provide the most mature regulatory evidence, while investigational programs remain separated by trial phase, delivery route, endpoint maturity, and safety follow‐up. Local in vivo programs such as subretinal EDIT‐101 illustrate both the feasibility and the limitations of ocular delivery in non‐dividing retinal cells, and systemic liver‐directed approaches such as TTR‐ or PCSK9‐targeting LNP‐mediated editing currently rely largely on early‐phase safety, pharmacodynamic, or biomarker outcomes. Safety‐limited programs must also be considered: VERVE‐101, an in vivo base‐editing program targeting PCSK9, showed LDL‐C and PCSK9 reductions in early data, but enrolment in the Heart‐1 study was paused in 2024 after a Grade 3 ALT elevation and Grade 3 thrombocytopenia in a treated participant. Accordingly, approved therapies, active clinical trials, paused or safety‐limited programs, and preclinical platforms should be separated when evaluating translational readiness (Figure 8).

### 9.2 Translational experience of ex vivo editing in haematological genetic diseases

Haematological genetic diseases were the first to enter clinical application, which is closely related to the characteristics of target cells and the treatment workflow. Regulatory and clinical data indicate^7^, ^91^, ^92^ that haematopoietic stem cells can be collected, subjected to ex vivo editing, quality control, and reinfusion, and can reconstitute the haematopoietic system after conditioning, thus making them suitable for forming a relatively standardized cell therapy pathway. For sickle cell disease and β‐thalassemia,^91^, ^92^, ^93^ CRISPR strategies do not necessarily require repairing each HBB mutation; instead, therapeutic effects can be achieved through editing of erythroid regulatory elements to increase fetal haemoglobin expression, thereby achieving functionally equivalent compensation at the mechanistic level. This approach exemplifies a function‐compensating editing concept, namely achieving therapeutic effects by reshaping disease‐related regulatory networks rather than relying solely on correcting pathogenic mutations in situ.

The feasibility of ex vivo editing in the haematopoietic system does not imply it is ready for widespread implementation.^35^, ^91^, ^92^ Regulatory and clinical practice data indicate that such therapies typically require haematopoietic stem‐cell collection, ex vivo editing, release testing, myeloablative conditioning, cell reinfusion, and long‐term monitoring, creating a complex treatment pathway with high demands on accredited centres. Myeloablative conditioning is particularly burdensome because it may involve acute toxicity, infection risk, hospitalization, fertility concerns, and late effects that must be weighed against therapeutic benefit. Cell production costs, scheduling constraints, manufacturing failure risk, reimbursement uncertainty, and long‐term registry participation may further limit real‐world adoption. Haematologic diseases therefore provide the clearest translational template for CRISPR therapy, but their experience cannot be directly extrapolated to genetic diseases in which target tissues are not collectable or reinfusable, or where pathological networks are more complex.

### 9.3 Challenges of in vivo editing in genetic diseases of the eye, liver, muscle, and nervous system

In vivo editing offers an alternative path for genetic diseases that cannot be addressed by cell reinfusion.^94^ Ocular diseases, owing to localized administration, relatively enclosed tissue environment, and well‐defined functional endpoints, have early on become a key scenario for in vivo CRISPR research; clinical registration data show that EDIT‐101, delivered subretinally, explores therapeutic potential for CEP290‐associated Leber Congenital Amaurosis 10 (LCA10). The liver, with its abundant blood flow, relative accessibility for delivery, and the hepatic origin of pathological mechanisms in various genetic metabolic diseases, is considered a major target organ for systemic in vivo editing; NTLA‐2001 registration data suggest that LNP‐mediated delivery of CRISPR components for Transthyretin (TTR) gene editing represents a key direction for clinical translation of in vivo editing.

The bottlenecks of in vivo editing vary across target tissues.^94^ From the translational perspective, ocular diseases highlight the advantages of local delivery but also reveal constraints related to subretinal administration, limited target‐cell accessibility, non‐dividing photoreceptors, and modest sample sizes. Liver diseases are more suitable for testing systemic LNP delivery and biomarker responses, whereas muscle and nervous‐system diseases foreground challenges posed by large tissue volume, broad cell distribution, biological barriers, and cellular complexity. Retinal, muscular, and neural tissues also differ in turnover rate and reversibility after developmental damage, so molecular editing or biomarker reduction should not be assumed to equal durable clinical benefit. For in vivo editing, unintended events are generally difficult to correct through cell screening as can be done in ex vivo editing. The key challenge is therefore not merely increasing editing efficiency, but achieving controllable repair in the correct tissue, correct cell type, and within an acceptable safety window.

### 9.4 Efficacy evaluation, long‐term follow‐up, ethical oversight, and real‐world application issues in clinical translation

The clinical value of CRISPR therapy for genetic diseases must be assessed collectively through durability of efficacy, functional improvement, and patient benefit. Regulatory data show that the indication for Casgevy (exa‐cel) in sickle cell disease is associated with recurrent vaso‐occlusive crises, while in β‐thalassemia it is associated with reduction in transfusion dependence; such haematological endpoints are relatively clear and facilitate efficacy evaluation. In contrast, clinical endpoints in genetic diseases of the nervous system, muscular system, and retina are more complex, often requiring a comprehensive assessment incorporating functional scores, imaging indicators, biomarkers, quality of life, and disease progression rate. Because many genetic diseases are rare diseases, clinical studies also frequently face challenges such as limited sample size, incomplete natural history data, difficulty in setting up control groups, and insufficient endpoint extrapolation. Therefore, patient selection, baseline stratification, natural history data, and the appropriateness of surrogate endpoints can directly impact efficacy judgment.

Regulatory practice typically emphasises that^95^ long‐term follow‐up and real‐world risk monitoring are important dimensions that distinguish gene editing therapy from conventional pharmacotherapy. FDA guidance documents indicate that gene therapy products may pose delayed risks, and thus long‐term follow‐up observation can become an integral component of risk monitoring for some products. For ex vivo HSC editing, the long‐term haematopoietic contribution of reinfused cells, clonal stability, immune responses, and potential tumour risk; for in vivo editing, attention is needed to delivery‐related immune responses, off‐target tissue editing, attenuation of editing effects, and delayed tissue damage. Therefore, long‐term follow‐up is used not only to confirm whether efficacy is sustained, but also to identify low‐frequency, delayed, or tissue‐specific safety risks.

Ethical, regulatory, and accessibility issues also influence the clinical translation of CRISPR‐based therapies for genetic diseases. For genetic diseases with childhood onset or rapid progression, early intervention may offer greater opportunities for functional preservation, yet it raises ethical concerns involving informed consent in minors, guardian decision‐making, long‐term risk assumption, and future autonomy. For individualized or small‐batch editing products, traditional drug evaluation frameworks may inadequately address requirements in design, manufacturing, release, batch consistency, and long‐term follow‐up. Therefore, an integrated regulatory approach incorporating product characteristics, editing mechanisms, delivery systems, and patient‐population risk is needed. Personalized cell therapies also depend on specialized treatment centres, manufacturing systems, long‐term registries, and payment mechanisms; high costs and regional disparities in medical resources directly affect treatment accessibility.^96^ Consequently, real‐world translation relies not only on efficacy and safety but also on the synchronous establishment of patient screening processes, ethical review mechanisms, regional treatment centre capacity, long‐term registries, and payment mechanisms (Table 4).

**TABLE 4:** Translational pathways, regulatory status, implementation thresholds, and real‐world barriers for CRISPR‐based treatment of genetic diseases

Pathway/program	Disease/indication	Regulatory status/clinical stage	Editing target or delivery mode	Representative efficacy outcome	Representative safety concern	Manufacturing/access barrier
Ex vivo HSC editing: Casgevy (exa‐cel)^91^	Sickle cell disease	Approved: FDA 2023 for ≥12 years with recurrent VOCs; FDA expanded label in 2026 to younger patients; EMA conditional authorization 2024	Autologous CD34+ HSPCs; CRISPR‐Cas9 editing of BCL11A erythroid enhancer; reinfusion after conditioning	VOC reduction/elimination; fetal haemoglobin induction; durable haematopoietic reconstitution	Myeloablative‐conditioning toxicity; secondary malignancies; clonal haematopoiesis; infertility; late haematopoietic effects; insertional/genotoxic risk if applicable	Cell collection, individualized manufacturing, conditioning, cryopreservation, specialized centres, high cost, and reimbursement
Ex vivo HSC editing: Casgevy (exa‐cel)^92^	Transfusion‐dependent β‐thalassemia	Approved/authorized in major regions for eligible patients; regulatory indication depends on age, transplant suitability, and regional label	Autologous CD34+ HSPCs; BCL11A enhancer editing to increase fetal haemoglobin	Transfusion independence; haemoglobin restoration; erythroid compensation	Conditioning‐related infertility/secondary cancers, clonal dynamics, infection, and long‐term reconstitution risk	Autologous manufacturing, patient selection, clean‐room capacity, treatment‐centre availability, and payment barriers
Local in vivo editing: EDIT‐101^97^	CEP290‐associated inherited retinal degeneration	Safety‐limited/halted or discontinued program; early‐phase ocular editing experience	Subretinal delivery of CRISPR‐Cas9 to photoreceptors	Exploratory visual‐function and retinal‐response endpoints	Local inflammation, ocular procedure risk, limited target‐cell accessibility, and uncertain durability	Specialized surgery, small eligible population, local delivery constraints, and limited scalability
Systemic in vivo editing: NTLA‐2001^98^	Transthyretin amyloidosis/cardiomyopathy	Investigational clinical program	LNP delivery of CRISPR‐Cas9 components targeting hepatic TTR	Serum TTR reduction; cardiac biomarkers and functional status	Infusion reactions, hepatic safety, immune response, and non‐target tissue exposure	Systemic LNP delivery, dose‐safety window, monitoring burden, and broad‐access implementation
Systemic in vivo editing: NTLA‐2002^99^	Hereditary angioedema	Investigational clinical program	LNP delivery of CRISPR‐Cas9 components targeting KLKB1	Reduced angioedema attacks; kallikrein suppression; durable biomarker response	Irreversibility of in vivo editing, immune response, and long‐term KLKB1 suppression	Dose selection, long‐term follow‐up, affordability, and regional trial access
Systemic in vivo base editing: VERVE‐101	Heterozygous familial hypercholesterolemia/ASCVD risk	Safety‐limited program; Heart‐1 enrolment paused/adjusted after serious laboratory abnormalities	LNP delivery of adenine base editor targeting PCSK9 in liver	LDL‐C and PCSK9 reduction as early biomarker response	Grade 3 ALT elevation and thrombocytopenia reported; hepatic/immune safety and off‐target base‐editing concerns	Early‐phase evidence, systemic delivery, dose escalation, monitoring, and uncertain long‐term benefit‐risk balance
Long‐term follow‐up/pharmacovigilance^95^	Approved and investigational gene/cell therapies	Required post‐authorization or trial‐linked surveillance	Registries, risk‐management plans, and long‐term monitoring systems	Durability of efficacy and delayed adverse‐event detection	Secondary malignancies, clonal expansion, immune events, late conditioning effects, and functional decline	Cross‐centre consistency, data completeness, patient adherence, and registry infrastructure
Real‐world access/payment framework^100^	High‐cost genetic medicines	Implementation barrier across approved and investigational products	Manufacturing networks, treatment centres, pricing and reimbursement models	Scaled treatment delivery and equitable long‐term benefit	Unequal benefit distribution and incomplete long‐term surveillance	High cost, centralized treatment, payer negotiation, cold chain, workforce capacity, and limited access in lower‐ and middle‐income settings

*Note*: Superscript numbers indicate representative references for the corresponding category. Regulatory labels and clinical stages should be rechecked immediately before submission because approvals and trial status can change.

### 9.5 Clinical implementation, CMC, manufacturing scalability, and real‐world access

Clinical translation also depends on implementation capacity rather than editing efficacy alone. Chemistry, Manufacturing, and Controls (CMC) should therefore be treated as part of translational feasibility, not as post‐approval logistics. Genome‐editing products require reproducible raw materials, GMP‐compatible editing reagents, validated release assays for identity, potency, purity, sterility, vector or RNA quality, editing outcome distribution, off‐target or structural‐variant risk, and batch‐to‐batch consistency. For autologous cell therapies, individualized manufacturing, cryopreservation or cold‐chain transport, product scheduling, and product failure risk may affect whether eligible patients can actually receive therapy. For in vivo products, scalable formulation, tissue targeting, stability, pharmacovigilance infrastructure, and regulatory comparability after manufacturing changes are decisive for broader implementation. High treatment cost, complex reimbursement, centralized treatment networks, and limited manufacturing or cold‐chain capacity may further restrict access, particularly in lower‐ and middle‐income settings. Table 4 should therefore distinguish regulatory status or clinical stage, representative efficacy outcomes, representative safety concerns, and manufacturing or access barriers for each translational pathway (Table 4).^97^, ^98^, ^99^, ^100^

### 9.6 From ‘editable’ to ‘treatable’: core bottlenecks in future clinical translation

CRISPR technology has enabled the rewriting of various types of genetic variants, but ‘editable’ does not equate to ‘treatable’. Clinical translation data indicate^7^, ^57^ that therapeutic editing must simultaneously meet requirements of clear target mechanisms, appropriate editing tools, feasible delivery routes, measurable functional recovery, controllable safety risks, and clinically meaningful benefits. Ex vivo editing offers advantages in cell detectability, selectability, and quality control, whereas in vivo editing benefits from direct action on diseased tissues. However, both face practical constraints in delivery efficiency, immune responses, cost burden, and long‐term safety. Thus, the key to clinical translation lies not merely in improving editing efficiency, but in ensuring that editing outcomes translate into sustained benefits in real‐world tissues and clinical settings.

Future clinical translation will likely depend more on the maturation of systemic platforms than on breakthroughs in single editing tools or delivery technologies.^26^, ^57^ Based on current research, CRISPR‐based therapy for genetic diseases requires the establishment of a closed‐loop system encompassing mutation diagnosis, tool selection, delivery implementation, functional assessment, safety follow‐up, and real‐world benefit evaluation. For monogenic diseases, the critical issue is improving the accuracy, safety, and accessibility of mutation correction; for complex or multi‐tissue disorders, a more cautious assessment of the risk–benefit ratio of multi‐target editing, regulatory editing, and combination therapies is necessary. Therefore, the future focus of translation will shift from merely proving technical feasibility to defining the thresholds in delivery, safety, manufacturing, follow‐up, and accessibility that must be crossed to progress from demonstrative therapies to broadly applicable treatment platforms.

## 10 CONCLUSION

The emergence of CRISPR‐Cas9 and related precision editing modalities is reshaping the investigation of genetic diseases by enabling the reconstruction, correction, or modulation of pathogenic mutations in their endogenous genomic context. This capability shifts emphasis from simply associating variants with disease towards establishing causal functional roles. Within this framework, genetic diseases can be seen not as fixed categories defined by mutation lists and clinical phenotypes, but as systems in which editing tools dissect the mechanistic chain that likely extends from pathogenic mutations through disruptions in gene function and aberrant cellular states to tissue‐level manifestations. CRISPR‐Cas9 is therefore positioned as a mechanistic link between the identification of pathogenic mutations, the dissection of gene function, and the design of precise repair approaches.

The diversification of precision editing tools has expanded the strategic options for confronting pathogenic mutations. These platforms are not arranged in a simple linear hierarchy; instead, each is preferentially suited to particular mutation architectures, intended functional outcomes, and inherent safety constraints. Classic CRISPR‐Cas9 editing via double‐strand breaks likely remains advantageous for gene knockout, mutation simulation, and partial sequence repair, whereas base editing may prioritize the precise rewriting of single‐nucleotide changes. For a subset of small insertions, deletions, and complex variants, prime editing potentially offers a more adaptable repair pathway, and the broadening of target accessibility and safety margins appears further advanced by Cas protein variants and non‐double‐strand‐break strategies. Collectively, these patterns suggest that achieving precise therapy for genetic diseases does not depend on forcing a single editing tool to fit all scenarios; rather, success likely hinges on matching the editing methodology to the specific mutation type, its functional consequences, the characteristics of the target tissue, and the established risk boundaries.

In the clinical translation arena, CRISPR technology has demonstrated proof‐of‐concept therapeutic feasibility in a limited set of genetic disease indications, yet broader implementation likely still faces substantial hurdles in delivery efficiency, safety assessment, long‐term follow‐up, treatment costs, and real‐world accessibility. The experience gained from ex vivo editing in haematological genetic diseases offers a valuable paradigm; however, for in vivo applications and disorders affecting complex tissues, more robust evidence of functional recovery and long‐term safety data are required before generalizable conclusions can be drawn. These current constraints suggest that the enduring value of CRISPR precision editing for genetic diseases may ultimately depend on its ability to establish a coherent, verifiable, regulatable, and generalizable therapeutic logic—one that integrates mechanistic disease classification, tool adaptation, delivery execution, functional evaluation, and long‐term safety monitoring into an interconnected framework. Limitations in each of these domains could delay clinical adoption, underscoring the need for systematic strategies that address the full translational pipeline.

## AUTHOR CONTRIBUTIONS

Z.W.: conceptualized the review, collected and analyzed the literature, prepared figures and tables, and drafted the manuscript. J.S.: supervised the work, reviewed and revised the manuscript. All authors read and approved the final manuscript.

## CONFLICT OF INTEREST STATEMENT

The authors declare no conflict of interest.

## FUNDING INFORMATION

The authors have nothing to report.

## CLINICAL TRIAL REGISTRATION

The authors have nothing to report.

## ETHICS STATEMENT

The authors have nothing to report.

## Supporting information




**Supporting File**: ctm270764‐sup‐0001‐figureS1.pdf

## Data Availability

All data generated or analyzed during this study are included in this article and/or its supplementary material files. Further inquiries can be directed to the corresponding author.
