# Supplementary material for: CRISPR‐Cas9 and precision editing technologies linking functional genomics to clinical translation in genetic diseases
Source: Clin Transl Med. 2026 Sep 24;16(9):e70764. doi: 10.1002/ctm2.70764 (PMC13602823; doi:10.1002/ctm2.70764)
Supplement: Supplementary file 1 [file CTM2-16-e70764-s001.pdf]

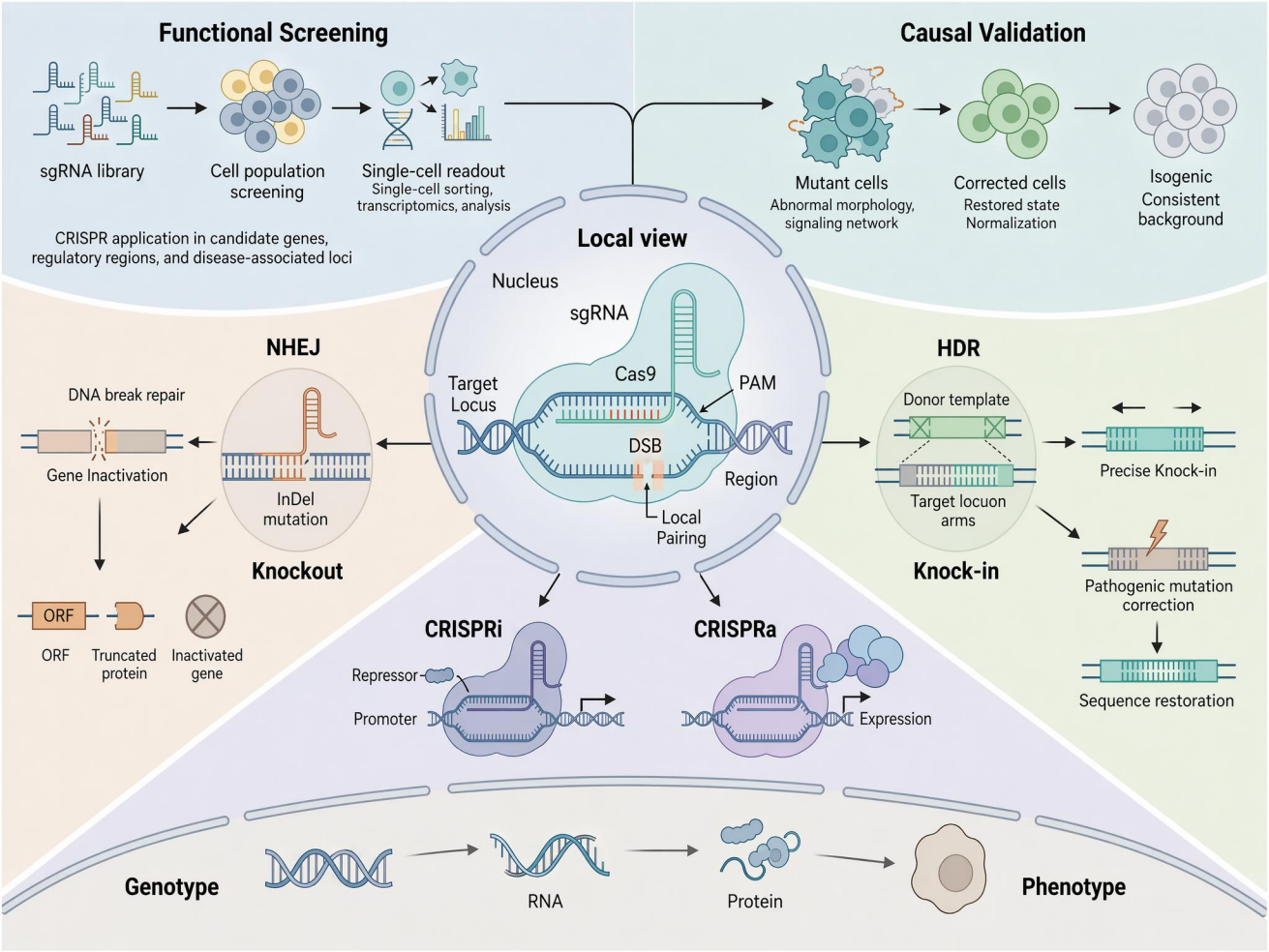

Functional Screening
sgRNA library
Cell population screening
Single-cell readout
Single-cell sorting, transcriptomics, analysis
CRISPR application in candidate genes, regulatory regions, and disease-associated loci
Causal Validation
Mutant cells
Abnormal morphology, signaling network
Corrected cells
Restored state
Normalization
Isogenic
Consistent
background
Local view
Nucleus
sgRNA
Cas9
Target Locus
PAM
DSB
Region
Local Pairing
NHEJ
DNA break repair
Gene Inactivation
InDel mutation
Knockout
ORF
ORF
Truncated protein
Inactivated gene
HDR
Donor template
Target locuon arms
Knock-in
Precise Knock-in
Pathogenic mutation correction
Sequence restoration
CRISPRi
Repressor
Promoter
CRISPRa
Expression
Genotype
RNA
Protein
Phenotype
